# Robotic-assisted gait training for spinal cord injury neuropathic pain: A systematic review

**DOI:** 10.1080/10790268.2025.2503049

**Published:** 2025-06-06

**Authors:** Conor White, Samantha Klus, Wing Hai Ho, Lily Barrett, Orlaith Doherty, Olive Lennon

**Affiliations:** School of Public Health, Physiotherapy and Sports Science, University College Dublin, Dublin, Ireland

**Keywords:** Robotic assisted gait training, Spinal cord injury, Neuropathic pain, Exoskeleton, Neurorehabilitation

## Abstract

**Context:**

Neuropathic pain (NP), can be a debilitating consequence of spinal cord injury. Robotic-assisted gait training (RAGT) is an effective rehabilitation tool, but emerging evidence suggests it may prove an effective treatment for NP post-SCI.

**Objective:**

This systematic review aims to synthesize the available evidence examining RAGT for pain reduction post-SCI, focusing on NP.

**Methods:**

Six databases were searched from inception to February 18th 2025, using search strings addressing: SCI and RAGT. No date or study type restrictions applied. Studies with SCI populations, delivering RAGT and measuring pain outcomes were included. Meta-analyses were conducted for pain intensity, pain interference (PI) and HRQoL outcomes. Certainty of evidence (COE) was assessed using GRADE criteria.

**Results:**

Thirty two studies (n = 567) (6 RCTs) met eligibility criteria. No RCT mandated pain as an inclusion criterion. Meta-analysis of 6 RCTs (n = 174) identified no effect for RAGT over other interventions for outcomes of overall pain intensity (SMD = 0.03 [−0.40, 0.46]; P = 0.90), non-specified pain-type intensity (SMD = 0.08 [−0.39, 0.55]; P = 0.74) and in one RCT reporting NP intensity (SMD = −0.46 [−1.61, 0.70]; P = 0.44). Meta-analysis identified no difference between RAGT and comparators for outcomes of HRQoL (3 RCTs (n = 69): SMD = 0.15 [−0.33,0.62]; P = 0.54) and PI (2 RCTs (n = 53): SMD = −0.1 [−0.64,0.44]; P = 0.71). Very-low COE for pain intensity & PI and low COE for HRQoL were assigned.

**Conclusions:**

Very limited studies and inconclusive evidence supports RAGT for the reduction of NP post-SCI. Future RCTs of RAGT that recruit patients with moderate-to-severe NP at baseline are required.

## Introduction

Pain can be a devastating consequence associated with spinal cord injury (SCI), with evidence suggesting that it is experienced by as many as 61% of this population (1). Neuropathic pain (NP), caused by a lesion or disease to the somatosensory nervous system (2), has a reported pooled prevalence of 53% in SCI (3). It is more common below the level of lesion, in patients with tetraplegia, older patients and at 1 year post injury (3). Mechanisms hypothesized for NP after SCI include neuronal hyperexcitability (central and peripheral sensitization) and corticothalamic maladaptive neuroplasticity (4). Additionally, NP symptom severity post-SCI is associated with a combination of residual spinothalamic tract (STT) function below the injury level and catastrophizing pain-coping mechanisms (5).

High pain intensity and pain interference levels and significantly poorer health-related quality of life (HRQoL) are associated with NP in comparison to other pain phenotypes after SCI (6,7). Individuals experiencing NP frequently describe it as their most persistent and unmet health issue (8,9). The intensity of NP likely increases over time, and it frequently becomes a chronic and life-long condition (10). While the mainstay of NP treatment after SCI is pharmacotherapy, (11), severe pain remains refractory to these treatments in two-thirds of sufferers (12). Significant side-effects of medications are common including in the central nervous system, which are often intolerable (13). These, together with fear of medication dependency, often result in poor adherence to pharmacological regimens leading to a call for non-pharmacological treatment options for people with NP after SCI (14,15). The mechanistic effect of sensorimotor stimulation on NP amelioration stems from Phantom Limb Pain research (PLP) and demonstrates reversal of cortical dysfunction in the primary somatosensory cortex (16). In NP after SCI, mechanistic- based research has largely centered on virtual reality and imagery-based walking (17,18). However, few actual sensorimotor intervention studies have been conducted to date (19). Little is understood about the potential role of robotic-assisted gait training (RAGT) for the reduction of SCI NP despite compelling pre-clinical gait studies (20–22). While RAGT is well established as a rehabilitation tool post-SCI, the focus of research has remained on gait-related outcomes and secondary outcomes addressing cardiovascular fitness, bladder/bowel function and spasticity (23–26).

The primary aim of this study is to synthesize all available evidence for the use of RAGTs in the management of SCI pain with a specific focus on NP. Secondary aims consider the effect of RAGT on pain interference and HRQoL after SCI.

## Methods

### Definitions

This systematic review is structured in accordance with the Preferred Reporting Items for Systematic Reviews and Meta-Analyses (PRISMA) 2020 Guidelines (27) and is registered with the International Prospective Register of Systematic Reviews (PROSPERO: CRD42018114431). See Supplementary Tables 9–10 (Tables S9, S10) for completed PRISMA checklists.

It was anticipated that most studies would fail to classify pain after SCI by phenotype, as the reporting of NP after SCI has previously lacked consistency in the literature (2). Thus, the population of interest for this review was chosen as individuals with any SCI-related pain. In the case of studies that did not define pain phenotype, non-specified pain intensity was recorded. In studies that defined pain phenotype, they were recorded as reported by the authors.

The intervention of interest was RAGT using an end-effector or exoskeleton device and the comparators were other exercise interventions, usual care, or sham interventions. The primary outcome was pain intensity. The secondary outcomes were pain interference and health-related quality of life (HRQoL).

### Search strategy

Guided by a liaison librarian, a comprehensive search strategy was created to identify studies reporting RAGT after SCI. Although pain, and specifically neuropathic pain, was the primary focus of this systematic review, it was not included as a search term in the electronic database search strategy. This broad and inclusive approach was taken to ensure that studies that reported pain as a secondary outcome or a subsequent finding (not reported in the abstract or keywords) would not be missed.

Two search strings were created to describe the two search topics of interest: RAGT and SCI. Medical Subject Headings (MeSH) or Controlled Vocabulary, appropriate to each database were employed, with the addition of free text. Variations of keywords for each independent search string were combined using the Boolean operator OR. The two search strings (RAGT and SCI) were combined using the Boolean operator AND. See Supplementary Table 8 (Table S8) for full details of search strategies for each database.

Six electronic databases were searched by title and abstract from their inception to February 18th 2025: PubMed, Embase, CINAHL, Scopus, Web of Science, and Cochrane Library. Additional studies were identified from the reference lists of review articles. No limitations were applied to any database in the initial search.

### Eligibility criteria

Predefined criteria for inclusion in the review were: adults (age 18 and above); a primary intervention of RAGT or including RAGT; and outcomes (primary or secondary) that included pain intensity, pain interference or HRQoL. Where a study did not list pain as an outcome measure of interest, but the authors reported pain as a subsequent or incidental finding, these studies were included. Studies reporting pain as an adverse event only were excluded.

Exclusion criteria included non-English language studies, studies where the full text was unavailable (*e.g.* conference abstracts), pre-clinical/animal studies, paediatric studies, and studies investigating other neurological conditions such as stroke or multiple sclerosis. As a nascent field, no limitations were placed on inclusion in the review by study type, with the exception of systematic reviews or meta-analyses. These were excluded; however, their reference lists were searched for eligible studies.

### Study selection

Search results from all databases were imported for screening to Covidence software (28). Search results were initially screened for eligibility by two independent reviewers by study title and abstract. If it was unclear at this level of screening whether a study assessed and reported pain as an outcome measure, an inclusive approach was taken whereby the article progressed to the next stage for more in-depth screening. At the final screening stage, full text articles were retrieved and read independently by two reviewers. Those that did not meet the predefined inclusion criteria were subsequently rejected, the remaining papers were included in our review. During all screening stages, where there was not full agreement between the two independent reviewers, a final consensus was reached through open discussion between the two reviewers and a third reviewer.

### Data extraction

Data were extracted to a standardized form in Excel software. The following information was extracted: 1. Study information (Author, date of publication, country of publication, study design) 2. Participant characteristics (sample size, mean age, sex, level of lesion, lesion type, extent of lesion according to AIS scale, aetiology of injury, time since injury) 3. Intervention characteristics (name of robotic device, intervention description, control description and intervention frequency, duration, and type) 4. Study outcomes and results (description of NP (where relevant), outcome measures, results, conclusions). If studies failed to report relevant outcome data or provided data in graph form only, authors were contacted to provide raw data. Where two or more RCTs were identified reporting the same outcome, their data were imported to REVMAN 5.4.1 software (29) for meta-analysis.

### Risk of bias assessment

The quality of the included studies was assessed by two independent reviewers, with conflicts resolved through discussion with a third reviewer. For RCTs the Cochrane Risk of Bias Tool 2 (ROB 2) (30) was used. This examines five domains that contribute to bias in randomized studies: the randomization process, deviations from intended interventions, missing outcome data, measurement of outcome and selective reporting of results. An overall risk of bias is then assigned to the study using the descriptors of high risk of bias, some concerns or low risk. For the remainder of quantitative studies, the Effective Public Health Practice Project (EPHPP) (31) was used. It examines eight components of quality in primary research, including where relevant selection bias, study design, confounding variables, blinding, data collection, withdrawals, data collection, and analyses. Results from the first 6 sections are compiled for a total study quality score of low risk of bias (no weak ratings), moderate risk of bias (1 weak rating), or high risk of bias (2 or more weak ratings). The EPHPP has shown fair inter-rater reliability between domains and excellent inter-rater reliability for the overall score (31).

The Critical Appraisal Skills Program (CASP) Tool was used to assess the risk of bias of qualitative studies (32). Endorsed by the Cochrane Qualitative and Implementation Methods Group, the CASP tool is commonly used to appraise health-related qualitative evidence. It is composed of 3 prompts: prompt A (five items) asks whether the results of the study are valid; prompt B (two items) asks what the results are; and prompt C (three items), asks if the results will help locally. Studies are classified as; (a) low risk of bias, therefore high quality, (studies scoring yes on 9 or 10 questions), (b) moderate risk of bias, therefore moderate quality (studies scoring yes on 5–8 questions) or (c) high risk of bias, therefore low quality (studies scoring yes on less than 5 questions) (33).

### Synthesis of results and analysis

Synthesis of results was undertaken using a two-pronged approach. Firstly, a meta-analysis was conducted, where feasible *i.e.* when data from two or more randomized controlled trials (RCTs) allowed pooling of outcome data related to pain intensity, pain interference, or HRQoL outcomes. Our analysis explored the effect of RAGT on overall pain intensity related to SCI, with sensitivity analysis examining its effect neuropathic pain intensity.

Where outcome measures for pain intensity, pain interference, or HRQoL in studies were the same, pooled mean differences with standard deviations were used in the meta-analysis. Where outcome measures for pain intensity or HRQoL studies differed across studies but measured the same construct, pooled standardized mean differences with 95% confidence intervals were used in the meta-analysis. Where median scores with interquartile range or minimum and maximum values were reported in the included manuscripts in relation to any of these outcomes, results were converted to mean and standard deviation scores using formulae outlined by Wan *et al.* (34). Where applicable, the standard deviation of the mean difference between scores was calculated according to the following formula (35,36):

$$\mathrm{SD}\;\mathrm{change}\;=\surd SD^{2}\mathrm{baseline}\;+\;SD^{2}\mathrm{final}\;-(2\;\times \;r\;\times \;\mathrm{SD}\;\mathrm{baseline}\;\times \mathrm{SD}\;\mathrm{final})\;(\mathrm{where}\;r\;=\;0.7)$$

where different scales were pooled with reverse scoring, scores were manually reversed with the formula “reverse score(x) = max(x) + 1 – x” prior to meta-analysis. As high heterogeneity between studies was anticipated, a random-effects model was used to calculate overall effect size (37). Statistical heterogeneity between studies was analysed using the I^2^ statistic. Meta-analysis was conducted using Revman 5.4.1 software (29).

Where meta-analysis was not possible, a narrative synthesis was conducted, supported by a best-evidence synthesis using Sackett’s level of evidence (38). Since the primary focus of this systematic review is NP after SCI, the best-evidence synthesis will ask the following questions:

1. What evidence currently supports RAGT as a treatment for those with neuropathic pain after SCI to reduce pain intensity levels?
2. What evidence currently supports RAGT to improve neuropathic pain interference following SCI?
3. What evidence currently supports RAGT to improve health-related quality of life for people with neuropathic pain following SCI?

Sackett’s level of evidence consists of five levels marked I to V and is categorized as follows:

Level I: (a) systematic review (with homogeneity) of RCTs, (b) individual RCT (with narrow CI).
Level II: (a) systematic review (with homogeneity) of cohort studies, (b) individual cohort study, including low-quality RCTs, (c) outcomes research.
Level III: (a) systematic review (with homogeneity) of case–control studies, (b) individual case control studies.
Level IV: case series (and poor-quality cohort and case–control studies).
Level V: expert opinion without explicit critical appraisal, or based on physiology, bench research or ‘first principles.

### Certainty of evidence assessment

Certainty of evidence identified in the review was established using the Grading of Recommendations, Assessment, Development, and Evaluations (GRADE) approach (39) for outcomes: pain intensity, HRQoL and pain interference comparing RAGT and comparator interventions.

## Results

### Study selection

**Figure 1:**
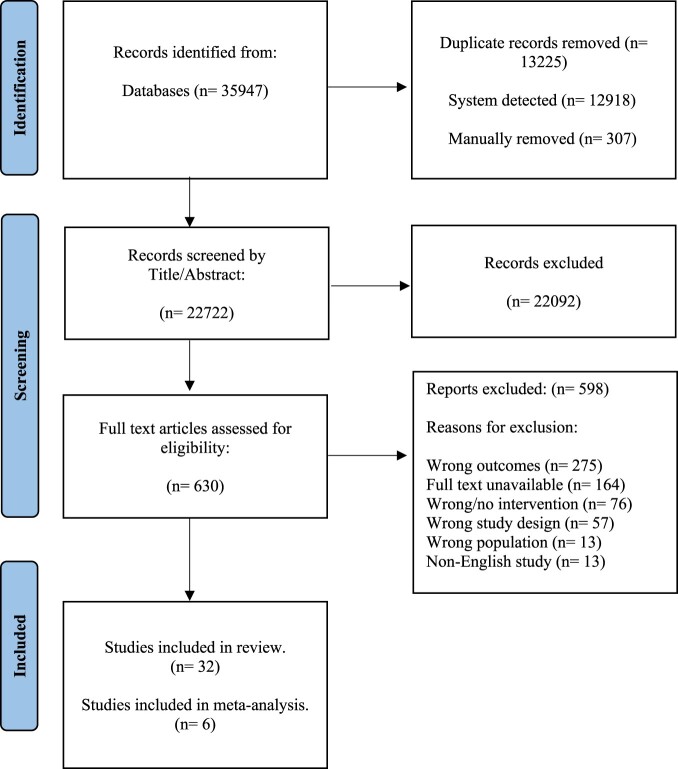
PRISMA flow diagram of study selection process

Figure 1 (PRISMA Flow Diagram) illustrates the study selection process. From a total of 18,430 unique papers identified, 31 studies met all eligibility criteria and were included in the final review. Main reasons for exclusion were incorrect intervention and incorrect outcome measures. Table 1 outlines the characteristics of each review study including author, study design, participant details, details of interventions, outcome measures and study results.

**Table 1:** Study characteristics

Author (year), design, country	Study population	Study setting	Participant pain profile at baseline	RAGT modality	Intervention	Comparator	Outcome measures	Results
Alcobendas-Maestro *et al.* (2012) (56), RCT, Spain	n = 75 (Int = 37; Con = 38) M/F: 47/28 T/NT: 36/39 LOI: Cer: 45; T/L: 30 C/I: 0/75 AIS: C,D TSI: 0.35 ± 0.22	Specialist SCI center	Low median pain score at baseline in both groups (0/10). Pain phenotype not specified.	Lokomat	8 weeks of 5x weekly sessions of RAGT walking alongside standard physiotherapy treatment. Session duration: 60 min	Conventional overground walking + standard physical treatment program	NS: VAS	**NS:** No significant within or between group differences (P > 0.05) **Int:** Pre: 1.7 ± 3.9 Post: 2 ± 4.6 MD: 0.3 ± 3.4 **Con:** Pre: 1.7 ± 3.9 Post: 1.2 ± 2.7 MD: −0.5 ± 2.8
Baunsgaard *et al.* (2018) (44), Prospective CS, Denmark	n = 52 M/F: 36/16 T/NT: 43/9 LOI: Cer: 14; T/L: 38 C/I: 25/27 AIS: A,B,C,D TSI: 3.2 ± 6.8	Nine European SCI Rehabilitation Centers	7 participants reported presence of NP. 15 participants reported presence of nociceptive pain.	Ekso GT	8 weeks of 3x weekly RAGT walking sessions. Session duration: 60 min	N/A	NOC: ISCIPBDS NP: ISCIPBDS PI: ISCIPBDS (All ISCIPBDS measures were qualitative reports of number of participants with pain taken 7 days prior to start of training and number of participants experiencing pain during training) HRQoL: ISCIQOLBDS	**PI:** ISCIPBDS: No significant change from pre to post intervention in number of participants experiencing pain interference. **NP:** N = 7 reported NP during intervention. **NOC:** N = 15 reported nociceptive pain during intervention. **HRQoL:** ISCIQOLBDS: No overall change in HRQoL pre to post intervention.
Benson *et al.* (2016) (54) Prospective CS, UK	n = 5 M/F: 5/0 T/NT: 5/0 LOI: Cer: 2; T/L: 3 AIS: A,C C/I: 3/2 TSI: 4.8 ± 6.2	Specialist SCI center	2 participants reported no pain 2 participants reported pain <1/10 intensity 1 participant reported pain <3/10 Pain phenotype not specified	ReWalk	19 weeks of 1-2x weekly sessions of exoskeleton progressive training covering 10 modules that incorporate functional activities, indoor/outdoor walking, and stairs management.	N/A	NS: VAS HRQoL: ATD-PA	**NS:** VAS: No significant change; Pre: 0.7 ± 1.1; Post: 0.9 **±** 1.2 **HRQoL:** ATD-PA: No significant change: Pre: 44 ± 7.3; Post: 48 ± 7.7
Cahill *et al.* (2018) (57) QS, Ireland	n = 4 M/F: 3/1 T/NT: 3/1 LOI: NR AIS: NR C/I: 2/2 TSI: 4.8 ± 3.6	Community Gym	N/A	Ekso	40–50 min interview with Research Question ‘'What are end users’ perspectives of using a robotic walking device within a gym setting?'	N/A	NS: QUAL	N = 4 subjectively reported decreased pain after intervention
Charbonneau *et al.* (2021) (58) QS, Canada	n = 9 M/F: 7/2 T/NT: 7/2 LOI: Cer: 1; T/L: 8 AIS: A,C,D C/I: 6/3 TSI: 0.18 ± 0.07	Tertiary referral, level 1 trauma center	N/A	Ekso GT	10–35 min interview examining the benefit of Esko Gait Training (∼22 sessions) in adjunct to existing in-patient rehabilitation programs in the acute phase.	N/A	NS: QUAL	Participants subjectively reported some improvement in pain, difficult to determine if this was the natural recovery process in acute injury.
Cinar *et al.* (2020) (59) Prospective CS, Turkey	n = 34 M/F: 23/11 T/NT: 27/7 LOI: Cer: 6; T/L: 28 AIS: A,B,C,D C/I: 17/17 TSI: 0.35 ± 0.08	Neurorehabilitation Hospital	Low median pain score. Pain phenotype not specified. Unable to ascertain exact pain intensity or pain interference scores (pooled)	Lokomat	5 weeks of twice weekly RAGT walking sessions in addition to conventional therapy which was provided 5 times weekly. The aim of the study was to examine if the benefits of RAGT differed for those complete v incomplete injuries.	N/A	NS: SF-36 BP subdomain PI: SF-36 BP subdomain HRQoL: SF-36	**NS/PI:** SF-36 BP: No significant change; **Complete group:** Pre 67.5 ± 21.7; Post 69 ± 21.7. **Incomplete group:** Pre: 67.5 ± 21.7, Post 69 ± 21.7 **HRQoL:** SF-36: Both groups displayed non-significant changes in all subdomains with exception of PF. Median Pre; Post Scores; (Complete/Incomplete) PF: 0;10/ 0;10 RP 100;100/ 100;100 GH: 52;52/ 52;56 V: 50;50/ 45;50 SF: 52;62/ 62;62 RE:100;100/ 100;100 MH: 47;52/ 48;56
Cinar *et al.* (2021) (60) RCT, Turkey	n = 37 (Int = 17; Con = 20) M/F: 15/22 T/NT: 29/8 LOI: Cer: 0; T/L: 37 AIS: A C/I: 37/0 TSI: 0.31 ± 0.46	Neurorehabilitation Hospital	Low median pain score. Pain phenotype not specified. Unable to ascertain exact pain intensity or pain interference scores (pooled)	Lokomat	8 weeks of twice weekly RAGT walking sessions. In addition to 5 times weekly conventional therapy.	8 weeks of 5 weekly sessions of conventional therapy: ROM, upper/lower stretching, strength, walking.	NS: SF-36 BP subdomain PI: SF-36 BP subdomain HRQoL: SF-36	**NS/PI:** SF-36 BP: No significant within or between group changes; **Int:** Pre: 61.3 ± 27.3; Post: 65.8 ± 26.8 MD: 4.5 ± 8.6 **Con:** Pre: 65.4 ± 22.4; Post: 68.9 ± 20.2 MD: 3.5 ± 7.4 **HRQoL:** SF-36: No significant overall effect for both groups. The only subdomain with a significant difference was physical activity which improved for both groups. Mean Pre; Post Scores; **PF:** Int: 17.1 ± 27.5; 21.8 ± 26.5/ Con: 17.0 ± 30.6; 22.5 ± 31.2 **RP:** Int: 63.2 ± 48.5;73.8 ± 35.0/ Con: 57.0 ± 45.8; 54.0 ± 46.4 **GH:** Int: 55.2 ± 19.6; 59.9 ± 18.1/ Con: 52.2 ± 19.8; 53.6 ± 20.4 **V:** Int: 48.1 ± 22.8; 51.1 ± 21.2/ Con: 45.7 ± 20.9; 49.3 ± 20 **SF:** Int: 68.6 ± 20.8; 69.3 ± 20/ Con: 60.7 ± 26.5; 60.3 ± 28.5 **RE:** Int: 66.6 ± 47.2; 68.4 ± 45.0/ Con: 56.5 ± 49.1; 61.2 ± 45.3 **MH:** Int: 47.1 ± 10.2; 51.9 ± 11.3/ Con: 46.7 ± 10.2; 46.6 ± 10.2
Cruciger *et al.* (2016) (40), CAS, Germany	n = 2 M/F: 1/1 T/NT: 2/0 LOI: Cer: 0; T/L: 2 AIS: A C/I: 2/0 TSI: 14.5 ± 6.4	Specialist SCI center	Authors specifically recruited participants with chronic NP of moderate to severe intensity (>4/10 NRS) Participant 1: 5/10 NP intensity at baseline Participant 2: 8/10 NP intensity at baseline	Hybrid Assistive Limb (HAL)	12 weeks of 5x times weekly bodyweight supported RAGT walking on a treadmill, in conjunction with conventional physiotherapy. Session duration: 30 minutes	N/A	NP: NRS PI: SF-36 BP subdomain HRQoL: SF-36	**NP:** NRS: Clinically meaningful reduction in overall pain severity; Pre: 6.5 ± 2.1; Post: 0.6 ± 0.1 **PI:** SF-36 BP subdomain: Significant improvement in both participants: Mean Pre; Post Scores (Participant 1/Participant 2) BP: 0;62/20;51 **HRQoL:** SF-36: Improvements in all subgroups of SF-36. Mean Pre; Post Scores (Participant 1/Participant 2) PF 5;10/ 35;95 RP 0;25/ 0;100 GH 10;30/ 30;80 VT 40;70/ 25;65 SF 12.5;62.5/ 12.5;75 RE 0;66.6/ 33;100 MH 40;36/ 44 68
Del-Ama *et al.* (2014) (61), CS, Spain	n = 3 M/F: 3/0 T/NT: 2/1 LOI: Cer: 0; T/L: 3 AIS: A,D C/I: 0/3 TSI: NR	Specialist SCI center	Pain reported at baseline was <1 mm on VAS. Pain phenotype not specified	Kinesis	1 week intervention with 4 sessions of hybrid gait training (EMS controlled).	N/A	NS: VAS (100 mm)	Non-significant change of 6.3 ± 17.3 across the participants.
Esquenazi *et al.* (2012) (62), Prospective CS, USA	n = 12 M/F: 7/5 T/NT: 11/1 LOI: Cer: 0; T/L: 12 AIS: NR C/I: 12/0 TSI: 7.4 ± 7.6	Laboratory	Not reported	ReWalk	8 weeks of 3x weekly sessions of RAGT along with gait and balance re-education. Session duration: 60–90 min	N/A	NS: VAS/QUAL	No quantitative VAS results reported N = 5 reported a combined 28 times that pain was reduced. N = 1 reported that pain was repeatedly increased immediately after the training intervention
Gant *et al.* (2018) (43), Prospective CS, USA	n = 8 M/F: 6/2 T/NT: 8/0 LOI: Cer: 0; T/L: 8 AIS: A,B C/I: 4/4 TSI: 10.4 ± 11.4	NR	Three of the eight participants reported pain at baseline. 1 participant reported 2 MSK pains and 1 below-level NP all of mild to moderate intensity. 1 participant reported 1 MSK pain and 1 at-level NP of moderate to severe intensity. 1 participant reported 2 MSK pains of mild to moderate intensity.	Lokomat	12 weeks of twice weekly progressive BWSTT walking along with upper extremity circuit training and the lower extremity FES cycle training.	N/A	MSK: ISCIPBDS (0-10 NRS) NP: ISCIPBDS (0-10 NRS) PI: ISCIPBDS (0-10 NRS)	**MSK/NP:** N = 3 experienced pain during the course of the study (BC02, BC03, BC07); **BC02:** reported 1 below-level NP, and two MSK pains. The area of the below-level NP and the moderate musculoskeletal shoulder pain dramatically decreased during the study. MSK pain intensity pre: 2/10 MSK pain intensity post: 2/10 NP intensity pre: 3/10 NP intensity post: 4/10 **BC03:** reported one constant at-level NP and one intermittent MSK pain. These pains remained constant with respect to both location and intensity during the study. MSK pain intensity pre: 5/10 MSK pain intensity post: 5/10 NP intensity pre: 7/10 NP intensity post: 7/10 **BC07:** reported two separate MSK pain problems at baseline and developed a 3rd during the study. MSK pain 1 intensity pre: 3/10 MSK pain 1 intensity post: 2/10 MSK pain 2 intensity pre: 5/10 MSK pain 2 intensity post: 2/10 MSK pain 3 intensity post: 7/10 **PI: BC02:** Pain interference with sleep was mostly minimal, but increased toward the end of the study. **BC03:** Pain interference with sleep remained relatively high throughout the study. **BC07:** Pain interference with sleep remained low throughout the study.
Hu *et al.* (2023) (63), RCT, China	n = 16 M/F: NR T/NT: NR LOI: Cer: 0; T/L: 16 AIS: A,B C/I: 12/4 TSI:±	NR	NR	Non-specified exoskeleton	8 weeks of 5x weekly sessions of Exoskeleton assisted walking along with conventional physiotherapy. Session duration: 40-50 minutes	Conventional physiotherapy only	NS: WHOQOL-BREF physical health domain HRQoL: WHOQOL-BREF	**NS:** WHOQOL-BREF physical health domain: No significant within or between group differences: **Int:** Pre: 9.8 ± 2.4 Post: 11.1 ± 2.9 MD: 1.3 ± 2.1 **Con:** Pre: 11.4 ± 2.3 Post: 11.8 ± 1.2 MD: 0.4 ± 1.7 **HRQOL:** WHOQOL-BREF: No significant within or between group differences: Psychological health domain: **Int:** Pre: 11.9 ± 2.0 Post: 12.1 ± 1.9 **Con:** Pre: 12.4 ± 1.9 Post: 13.3 ± 1.1 Social relationships domain: **Int:** Pre: 11.8 ± 1.6 Post: 12.2 ± 1.7 **Con:** Pre: 12.7 ± 2.3 Post: 12.7 ± 2.3 Environment domain: **Int:** Pre: 11.2 ± 2.6 Post: 11.1 ± 2.3 **Con:** Pre: 11.6 ± 1.9 Post: 11.8 ± 1.2 **Total MD** **±** **SD: Int:** 0.5 ± 1.8 **Con:** 0.4 ± 1.9
Juszczak *et al.* (2018) (55), CS, USA	n = 45 M/F: 37/8 T/NT: NR LOI: Cer: 0; T/L: 45 AIS: A,B,C C/I: 30/15 TSI: 3.9 ± 5.1	Specialist rehabilitation center	Mean pain intensity mild at baseline. Pain phenotype not specified.	Indego	25 sessions progressive RAGT walking over 8 weeks, along with functional skill acquisition in relation to walking.	N/A	NS: NRS HRQoL: SWLS	**NS:** NRS: No significant change (P > 0.05); Pre 1.1 ± 1.7 Post 0.9 ± 1.6 SWLS: Non-significant change (P > 0.05); Pre: 20.4 ± 8.0 Post: 21.3 ± 7.6
Khan *et al.* (2019) (45), CS, Canada	n = 12 M/F: NR T/NT: 12/0 LOI: Cer: 3; T/L: 9 AIS: A,C,D C/I: 6/6 TSI: 7.6 ± 8.1	University laboratory	6 participants reported low NP intensity at baseline (< 10 MPQ index) 3 participants reported higher NP intensity scores (>10 MPQ index)	ReWalk	12 weeks of 5x weekly progressive sessions of RAGT sit-to-stand, stand-to-sit, standing balance and walking along with transfer practice.	N/A	NS: NRS NP: MPQ index	**NS:** NRS: No significant change; Pre 2.0 ± 1.6; Post 1.2 ± 0.9 **NP:** MPQ: No significant change; Pre 9.2 ± 8.7; Post 10.7 ± 6.1
Khande *et al.* (2024) (49), Prospective Comparative Study, India	n = 30 (Int = 15; ComP = 15) M/F: 24/6 T/NT: 24/6 LOI: Cer: 0; T/L: 30 C/I: 30/0 TSI: Acute	University	Mean pain intensity severe at baseline. Pain phenotype not specified	Lokomat	12 weeks of RAGT walking along with ROM, strengthening exercises and tilt table.	12 weeks of conventional physiotherapy. Participants stood and walked in parallel bars using KAFOs and completed ROM, strengthening exercises and tilt table.	NS: VAS (0-10) HRQoL: McGill QOL	**NS:** Significant improvements in both groups. No significant between group differences (P = 0.099). **Int: P** **=** **0.001** Pre: 7.2 ± 0.6 Post: 5.3 ± 0.6 **Comp: P** **=** **0.001** Pre: 7.3 ± 0.6 Post: 5.7 ± 0.6 **HRQoL:** Significant improvements in both groups. Significant between group difference in favor of intervention group (P = 0.0001) **Int: P** **=** **0.0001** Pre: 20.9 ± 2.4 Post: 56 ± 6.5 **Comp: P** **=** **0.0001** Pre: 19.5 ± 1.8 Post: 41.2 ± 3.7
Kim *et al.* (2021) (64), Prospective CS, South Korea	n = 10 M/F: 7/3 T/NT: NR LOI: Cer: 1; T/L: 9 AIS: A,B,C C/I: 7/3 TSI: 5.7 ± 4.8	Laboratory	Mean pain intensity mild to moderate at baseline. Pain phenotype not specified.	H-Mex	10 weeks, 3x weekly of transfer practice and RAGT walking over flat ground. Session duration: 60 minutes.	N/A	NS: SF-36 BP subdomain PI: SF-36 BP subdomain HRQoL: SF-36	**NS/PI: SF-36 BP:** No significant change; Pre 58.4 ± 23.0, Post 62.5 ± 19.4. **HRQoL:** SF-36: No significant overall change Mean Pre; Post Scores; **PF:** 19.5 ± 14.0; 15.0 ± 9.1 **RP:** 55.6 ± 31.1; 56.3 ± 27.5 **GH:** 62.0 ± 17.2; 65.5 ± 16.9 **V:** 53.8 ± 13.9; 61.9 ± 14.3 **SF:** 67.5 ± 24.4; 71.3 ± 26.4 **RE:** 64.2 ± 34.9; 67.5 ± 29.8 **MH:** 69.5 ± 15.4; 80.0 ± 11.6
Koljonen *et al.* (2021) (65), CS, Hong Kong/USA	n = 40 M/F: 28/12 T/NT: NR LOI: Cer: 0; T/L: 40 AIS: A,B,C C/I: 24/16 TSI: Chronic	Two specialist rehabilitation centers	Mean pain intensity mild at baseline (<2/10 NRS). Pain phenotype not specified.	SuitX Phoenix	20 sessions of transfer practice, balance training, RAGT walking with exoskeleton over flat ground. Session duration: 60 minutes.	N/A	NS: NRS	**NS: NRS:** No significant change; Pre: 1.5 ± 1.7, Post: 1.1 ± 1.4.
Kressler *et al.* (2014) (41), CSS, USA	n = 3 M/F: 2/1 T/NT: NR LOI: Cer: 0; T/L: 3 AIS: A C/I: 3/0 TSI: Chronic	University	3 participants reported moderate to severe NP intensity at baseline	Ekso	6 weeks, 3x weekly of over ground RAGT walking around an oblong track (24.4 m) using a walker for balance and an overhead tether for falls prevention. Participants progressed through Esko's three modes of walking based on walking quality and participant feedback. Session duration: 60 minutes.	N/A	NP: ISCIPBDS (0-10 NRS) PI: ISCIPBDS Sleep interference item (0-6 Likert Scale)	**NP: NRS:** 2 of the 3 participants experienced a clinically meaningful reduction (MCID = 30%) in pain from pre to post intervention **Participant 1:** Pre: 7/10; Post: 5/10 **Participant 2:** Pre: 7/10; Post 2/10 **Participant 3:** Pre: 4/10; Post: 5/10 **PI: ISCIPBDS:** 2 of the 3 participants experienced reductions in sleep interference from pre to post intervention: **Participant 1:** Pre: 6/6; Post: 5/6 **Participant 2:** Pre: 4/6; Post: 2/6 **Participant 3:**Pre: 3/6; Post: 3/6
Labruyere *et al.* (2014) (66), RCOS, Switzerland	n = 9 (Int = 5; Con = 4) M/F: 5/4 T/NT: 4/5 LOI: Cer: 5; T/L: 4 AIS: D C/I: 0/9 TSI: 4.2 ± 4.7	Specialist SCI center	1 participant reported no pain. 6 participants reported presence of below-level NP 4 participants reported presence of at-level NP 4 participants reported presence of MSK pain	Lokomat	4 weeks, 4x weekly of progressive RAGT walking. Session duration: 45 minutes.	4 weeks of lower limb strength training (10-minute warm up followed by 3 sets of 10–12 reps of 4–6 exercises at 70% MVC)	NS: VAS (100 mm)	**NS: VAS:** No significant within/between group changes: **Int:** Pre: 28.6 ± 3.0; Post: 24.1 ± 3.3, MD: −4.1 ± 2.5 **Con:** Pre: 29.6 ± 2.6; Post: 22.7 ± 3.7 MD: −6.9 ± 2.6
Martinez *et al.* (2018) (48) RCOS, USA	n = 12 (Int = 6; Con = 6) M/F: 9/3 T/NT: 10/2 LOI: Cer: 3; T/L: 9 AIS: A,B,C,D C/I: 1/11 TSI: 7.9 ± 7.3	Medical center	4 participants reported no NP at baseline 4 participants reported mild NP at baseline (<3 MPQ VAS) 4 participants reported moderate intensity NP at baseline (3-6 MPQ VAS)	Lokomat	10–15 weeks of 3–5 x weekly progressive RAGT walking. Session duration: 30 minutes There was a washout period of 6 weeks and then the groups crossed over into the other intervention.	10–15 weeks of 3–5 x weekly multimodal exercise program (Balance, skilled upper extremity exercises with BWS using the Lokomat).	NP: MPQ (VAS)	**NP: MPQ:** No significant within/between group changes: **Int:** Pre: 4.3 ± 6.0 Post: 2.8 ± 2.5 MD: −1.5 ± 4.6 **Con:** Pre: 2.7 ± 4.6 Post: 4.5 ± 7.8 MD: 1.8 ± 5.6
Mazzoleni *et al.* (2017) (50), CS, UK	n = 7 M/F: 5/2 T/NT: 7/0 LOI: Cer: 0; T/L: 7 AIS: A C/I: 7/0 TSI: NR	Specialist SCI center	Mean pain intensity at baseline was moderate intensity. Pain phenotype not specified.	Ekso	Training was composed of 2 phases: 20 training sessions (3x weekly) using FES cycling system followed by 20 training sessions (3x weekly) using Esko.	N/A	NS: NRS HRQoL: ISCIQOLBDS	**NS: NRS:** No significant change; Pre 5.1 ± 2.5; Post: 3.9 ± 3.1 **HRQoL: ISCIQOLBDS:** No significant change; Pre 20.9 ± 7.2; Post: 15.6 ± 11.8
Platz *et al.* (2016) (46), CS, Germany	n = 7 M/F: 5/2 T/NT: NR LOI: Cer: 0; T/L: 7 AIS: A,C C/I: 6/1 TSI: 11.4 ± 10.1	Specialist SCI center	NR	Non-specified exoskeleton	4–5 weeks of 5x weekly overground exoskeleton walking with individualized therapeutic exercise as indicated; sit to stand and reverse, standing balance, weight shifting training prior to training Session duration: 60 mins	N/A	NS: SF-12 BP NP: QUAL PI: SF-12 BP HRQoL: SF-12	***Raw Data Unavailable:*** **NS/PI:** SF-12 BP: No significant change. **NP:** N = 2 subjectively reported decreased NP during training. **HRQoL:** SF-12: No significant changes in overall score.
Sale *et al.* (2016) (67), CAS, Italy	n = 3 M/F: 2/1 T/NT: NR LOI: Cer: 0; T/L: 3 AIS: A,C C/I: 2/1 TSI: Chronic	Neurorehabilitation hospital	1 participant reported severe pain intensity at baseline 2 participants reported mild pain intensity at baseline. Pain phenotype not specified	Ekso	20 sessions, 3-4x weekly of rehabilitation mobility training (sit-to-stand, standing activities with parallel bars, stand-sit transfers, standing balance, and stepping skills) using different Ekso modes. Session duration: 50 minutes.	N/A	NS: VAS	**NS:** No significant change; Pre: 3.3 ± 4.0, Post: 3 ± 3.5
Sale *et al.* (2018) (68), Prospective CS, Italy	n = 8 M/F: 6/2 T/NT: NR LOI: Cer: 0; T/L: 8 AIS: A,B,C C/I: 3/5 TSI: Chronic	Neurorehabilitation hospital	Mean pain intensity mild at baseline. Pain phenotype not specified.	Ekso	20 sessions, 4-5x weekly of walking with exoskeleton device & front rolling walker (3 × 30 min training sessions prior to intervention). Session duration: 45 minutes.	N/A	NS: VAS	**NS:** No significant change; Pre 1.0 ± 2.8, Post 0.9 ± 2.5
Sawada *et al.* (2021) (42), CS, Japan	n = 19 M/F: 14/5 T/NT: 15/4 LOI: Cer: 10; T/L: 9 AIS: A,B,C,D C/I: 2/17 TSI: 6.7 ± 11	University	Mean pain intensity moderate at baseline. Pain phenotype not specified.	Non-specified voluntary driven exoskeleton over treadmill	20 sessions, 2-5x weekly of RAGT walking. Session duration: 60 minutes.	N/A	NS/PI: SF-36 BP subdomain NP: NPSI HRQoL: SF-36	**NS/PI:** SF-36 BP: No significant change: Pre: 44.7 ± 12.6 Post: 42.9 ± 12.2 **NP:** NPSI: No significant change: Pre: 14.5 ± 15.2 Post: 13.5 ± 13.8 **HRQoL:** SF-36: No significant change: Mean Pre; Post Scores; PF: −3.0 ± 10.4; −3.0 ± 8.4 RP: 35.6 ± 17.0; 36.8 ± 15.4 GH: 47.6 ± 11.8; 48.3 ± 10.5 V: 46.6 ± 9.0; 44.3 ± 10.6 SF: 37.0 ± 16.3; 38.7 ± 14.0 RE: 44.5 ± 10.3; 45.3 ± 13.1 MH: 49.0 ± 5.6; 47.6 ± 8.7
Shackleton *et al.* (2023) (51) RCT South Africa	n = 16 (Int = 8; Con = 8) M/F: 15/1 T/NT: 16/0 LOI: Cer: 16; T/L: 0 AIS: C,D C/I: 0/16 TSI: 10.6 ± 10.4	University	Mean pain intensity moderate at baseline. Pain phenotype not specified.	Ekso GT	24 weeks 3x weekly of exoskeleton walking. Session duration: 60 minutes	24 weeks 3x weekly of combined exercise program consisting of resistance, cardiovascular, and flexibility training in various positions as well as gait retraining, without a treadmill or robotic assistance Session duration: 60 minutes	NS: ISCIPBDS (0-10 NRS) PI: ISCIPBDS (Activity, Mood, Sleep domains) (0-10 NRS) HRQoL: ISCIQOLBDS	**NS:** ICIPBDS: No significant within/between group differences **Int:** Pre: 4.2 ± 2.7 Post: 5.7 ± 1.6 MD: 1.5 ± 3.4 **Con:** Pre: 4.0 ± 2.2 Post: 5.6 ± 1.5 MD: 1.6 ± 2.9 **PI: ISCIPBDS:** No significant between group differences in 3 domains (P = 0.61). Significant within group increase in pain interference in daily activity domain in both groups (P = 0.05) Mean Pre; Post Scores; MD (all ±SD) **Activity: Int:** 2.3 ± 1.8; 4.3 ± 1.9; 2.0 ± 2.9 **Con:** 2.4 ± 2.1; 4.8 ± 2.9; 2.4 ± 3.9 **Mood: Int:** 2.6 ± 2.8; 4.3 ± 2.0; 1.8 ± 3.8 **Con:** 2.3 ± 2.6; 3.4 ± 2.7; 1.1 ± 4.1 **Sleep: Int:** 3.1 ± 4.3; 3.1 ± 3.0; 0.0 ± 5.7 **Con:** 1.4 ± 1.5; 2.9 ± 2.1; 1.5 ± 2.8 **Total MD** **±** **SD: Int:** 1.3 ± 7.4 **Con:** 1.7 ± 6.3 **HRQoL: ISCIQOLBDS:** No significant between group differences in general QoL (P = 0.16) and psychological QoL (P = 0.26). Significant between group difference in physical QoL in favor of intervention group: Mean Pre; Post Scores; MD (all ±SD) **General: Int:** 6.3 ± 2.3; 8.6 ± 1.4; 2.4 ± 3.0 **Con:** 6.8 ± 2.3; 7.5 ± 1.6; 0.8 ± 3.1 **Psychological: Int:** 8.0 ± 1.7; 8.3 ± 1.8; 0.3 ± 2.7 **Con:** 7.1 ± 2.5; 7.8 ± 2.1; 0.6 ± 3.6 **Physical: Int:** 6.6 ± 1.7; 8.6 ± 2.0; 2.0 ± 2.3 **Con:** 6.5 ± 2.3; 7.1 ± 2.3; 0.6 ± 3.5 **Total MD** **±** **SD: Int:** 1.6 ± 4.6 **Con:** 0.7 ± 5.9
Stampacchia *et al.* (2016) (52), CS, Italy	n = 21 M/F: 17/4 T/NT: 14/7 LOI: Cer: 4; T/L: 17 AIS: A,B,C C/I: 12/9 TSI: 10.2 ± 8.7	Specialist SCI center	Mean pain intensity moderate at baseline. Pain phenotype not specified.	Ekso GT	1 session of exoskeleton assisted walking and sit to stand training. Session duration: 40 minutes.	N/A	NS: NRS	**NS: NRS:** Significant reduction in pain in those who reported pain at baseline (N = 12) (P < 0.05) Pre: 5.9 ± 0.6, Post: 2 ± 1.1
Tamburella *et al.* (2020) (53), CSS, Italy	n = 4 M/F: 4/0 T/NT: 3/1 LOI: Cer: 1; T/L: 3 AIS: D C/I: 0/4 TSI: 1.5 ± 1.1	Neurorehabilitation hospital	Mean pain intensity moderate at baseline. Pain phenotype not specified.	Achilles ankle exoskeleton with neuromuscular controller (NMC)	10 sessions, 3-4x weekly of ankle and knee movements with exoskeleton and NMC. Sit to stand practice, balance training, exoskeleton walking. Session duration: 40 minutes.	N/A	NS: VAS	**NS: VAS:** No significant change; Pre: 4.3 ± 2.6; Post: 2.3 ± 2.5
Van Dijsseldonk *et al.* (2020) (47), CS, Netherlands	n = 14 M/F: 7/7 T/NT: NR LOI: Cer: 0; T/L: 14 AIS: A,B C/I: 4/10 TSI: 9.4 ± 8.5	Community	Not reported	ReWalk	Authors assessed exoskeleton use in community dwelling SCI patients in terms of number of steps taken, purpose of use and location of use. Data collection took place over 2–3 weeks; Session duration: 50 minutes.	N/A	MSK/NP: QUAL	**MSK/NP:** N = 1 reported reduced neuropathic pain after exoskeleton use. N = 4 reported muscle or joint pain. N = 2 reported shoulder pain after exoskeleton use.
Van Nes *et al.* (2022) (69) CS, Netherlands	n = 21 M/F: 13/8 T/NT: NR LOI: Cer: 0; T/L: 21 C/I: 20/1 TSI: 9.7 ± 7.9	Neurorehabilitation center	Mean pain intensity mild to moderate at baseline. Pain phenotype not specified.	ReWalk	24 sessions 3x weekly of exoskeleton walking. Session duration: 90 minutes	N/A	NS: SF-36 BP subdomain PI: SF-36 BP subdomain HRQoL: SF-36	**NS/PI: SF-36 BP:** Significant improvement (P = 0.003) Pre: 63 ± 22 Post: 75 ± 16 **HRQoL: SF-36.** Significant improvement in sum score (P = 0.02) Pre: 571 ± 133 Post: 621 ± 90
Van Silfhout *et al.* (2020) (70), CS, Czech Republic	n = 6 M/F: 5/1 T/NT: NR LOI: Cer: 1; T/L: 5 AIS: A,B C/I: 4/2 TSI: 0.63 ± 0.25	Neurorehabilitation center	Mean pain intensity mild at baseline. Pain phenotype not specified.	Lokomat	6 sessions of RAGT walking over treadmill with varying degrees of body weight support. Session duration: 30 minutes.	N/A	NS: VAS	**NS:** **VAS:** Significant reduction in pain intensity; (P < 0.05) Pre: 2.5 ± 1.9, Post: 1.5 ± 1.8
Yildiz *et al.* (2024) (71) CS, Turkey	n = 25 M/F: 18/7 T/NT: NR LOI: Cer: 0; T/L: 25 AIS: A,B,C,D C/I: 12/13 TSI: 2.6 ± 1.66	University	Not reported	Lokomat	6 weeks of 3x weekly of RAGT walking over treadmill with varying degrees of body weight support. Session duration: 30 minutes.	N/A	NS: WHOQOL-BREF physical health domain HRQOL: WHOQOL-BREF	**NS: WHOQOL-BREF physical health domain:** Significant improvement from baseline to post-treatment (P < 0.001) Pre: 41.9 ± 10.2, Post: 49.7 ± 6.8 **HRQOL: WHOQOL-BREF:** Significant improvement in all domains (P < 0.001) General Health: Pre: 48.3 ± 11.7, Post: 57.3 ± 7.8 Psychological: Pre: 38.6 ± 9.6, Post: 45.9 ± 6.3 Social relations: Pre: 58.0 ± 14.1, Post: 68.8 ± 9.4 Environment: Pre: 64.2 ± 15.7, Post: 76.0 ± 9.0

Statistically significant difference is considered as P < 0.05 unless otherwise stated.

Time since injury (TSI) indicated in years.

AIS, Asia Impairment Scale; ATD-PA, Assistive Technology Device Predisposition Assessment; BP, Bodily Pain; BWSTT, Bodyweight Supported Treadmill Training; C: Complete; Cer: Cervical; Con, Control; CAS, Case Study; Comp: Comparator; CS, Cohort Study; CSS, Case Series; F, Female; FES: Functional Electrical Stimulation; GH, General Health; HRQoL, Health Related Quality of Life; I: Incomplete; Int, Intervention; ISCIPBDS, International Spinal Cord Injury Pain Basic Data Set; ISCIQOLBDS: Internation Spinal Cord Injury Quality of Life Basic Data Set; KAFO, Knee-Ankle-Foot Orthosis; LOI, Level of Injury; M, Male; MD: Mean Difference; MH, Mental Health; MPQ, McGill Pain Questionnaire; MSK, Musculoskeletal; N, Sample Size; N/A: Not Applicable; Noc, Nociceptive; NP, Neuropathic Pain; NPSI, Neuropathic Pain Symptom Inventory; NR, Not reported; NRS, Numerical Rating Scale; NS, Non-specified Pain; PF, Physical functioning; PI: Pain Interference; QS, Qualitative Study; QUAL: Qualitative; RAGT, Robotic Assisted Gait Training; RCOS, Randomized Cross-over Study, RE, Role emotional; ROM, Range of Motion; RP, Role physical; SD: Standard Deviation; SF, Social functioning; SF-12, Short Form Health Survey-12; SF-36; Short Form Health Survey-36; SWLS, Satisfaction with Life Scale; T/L: Thoracic/Lumbar; T/NT: Traumatic/Non-Traumatic; TSI: Time Since Injury; V, Vitality; VAS, Visual Analogue Scale; WHOQOL-BREF, World Health Organization Quality of Life Assessment- Abbreviated Version.

### Patient demographics (Figure 2)

#### Participant characteristics

A total of 567 participants were included across the 32 studies. 29 studies (n = 520) reported sex demographics. 363 (69.8%) were male and 150 (30.2%) were female. 31 studies (n = 548) reported ASIA impairment scale. 282 of the total study participants (51.5%) had a complete SCI (ASIA A), with 266 (48.5%) of the participants documented as having an incomplete SCI (ASIA B, C and D). 30 studies (n = 544) documented neurological level of injury. Of the total study participants, 112 (20.6%) had a documented cervical level SCI, the remaining 432 (79.4%) had thoracic or lumbar level SCI. 20 studies (n = 371) reported SCI aetiology: 278 participants (74.9%) suffered traumatic SCIs and 93 (25.1%) suffered non-traumatic SCIs. 28 studies (n = 522) provided data on time since injury; 23 (n = 438) quantified the time with a weighted mean (based on sample size) time since injury of 3.9 years +/- 5.8 years. Four studies described participants as being in a chronic stage of injury. One study described participants as being in an acute stage of injury.

**Figure 2:**
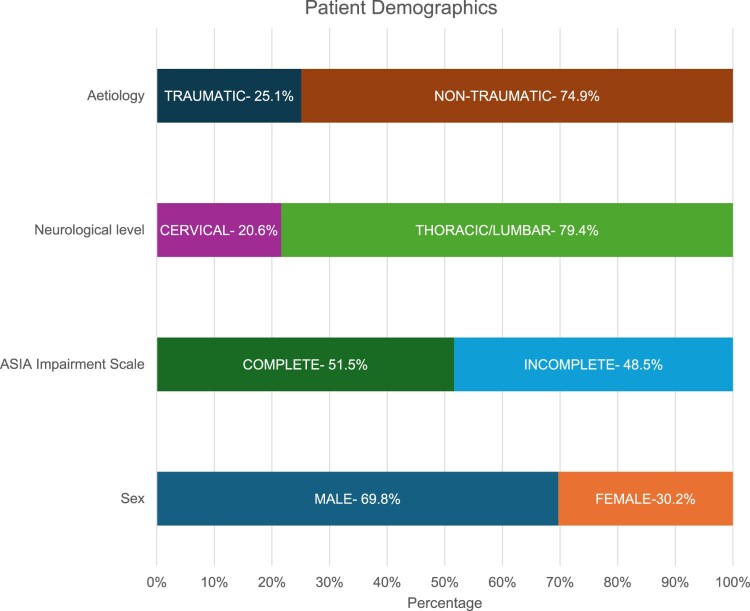
Patient demographics

#### Overview of studies and participant pain profiles

Of the 32 studies included in final review, the following study designs were identified:

- 19 cohort studies.
- Six RCTs (two using a cross over design).
- Four case series/case studies.
- Two qualitative studies.
- One prospective comparative study.

Nine studies (n = 129) described participants as having NP at baseline 2 case studies/case series (40,41); six cohort studies (42–47); 1 RCT (48).

Only 1 case study (n = 2) (40) included neuropathic pain at baseline as an inclusion criterion. The remaining 30 studies did not specifically recruit participants with pain of any phenotype at baseline and reported mean pain scores across all participants regardless of pain status at baseline. Seven studies (n = 83) (three case studies/case series (n = 9); one RCT (n = 16) and two cohort studies (n = 28); one prospective comparative study (n = 30)) reported moderate-to-severe pain intensity for participants at baseline (>4/10 and >6/10 on NRS or VAS) (40,41,49–53). Of these, only two case studies/case series reported moderate-to-severe neuropathic pain intensity for participants at baseline (40,41)

Seven studies (n = 160) (one RCT (n = 16); one case study (n = 2); four cohort studies (n = 137); one qualitative study (n = 5)) (40,42,44,51–55) assessed pain intensity as a primary outcome measure. The remainder of the studies assessed it as a secondary outcome measure.

### Interventions

29 studies (n = 532) reported the RAGT intervention duration. The weighted mean (based on sample size) duration of interventions was 7.7 weeks (range: 1–24 weeks). The weighted mean number of sessions for interventions reported across 28 studies (n = 505) studies was 25.6 (range: 1–72 sessions). The weighted mean session duration reported in 21 studies (n = 359) was 53.5 minutes (range: 30–90 minutes).

### RAGT modalities

The most commonly used RAGT modalities were the Lokomat, Ekso and ReWalk (Table 1).

### Study setting

The most common study settings were specialist SCI centers, neurorehabilitation hospitals and university laboratories (Table 1).

### Outcome measures

#### Non-specified pain intensity

Of the included studies, 28 assessed pain intensity where pain type was non-specified (17 cohort studies, five RCTs, three case series/case studies, two qualitative studies, one non-randomized trial). Significant heterogeneity in outcome measures used to assess non-specified pain intensity was evident. The most commonly used outcome measures were the Visual Analog Scale (VAS) (72), the Numerical Rating Scale (NRS) (73) and the bodily pain domain of the 36-item Short Form Survey (SF-36) (74) (Table 1 and supplementary Table 1 (Table S1)).

#### Neuropathic pain (NP) intensity

Nine of the included studies reported assessing NP intensity using neuropathic pain specific scales. The most commonly used outcome measures were the International Spinal Cord Injury Pain Basic Dataset (ISCIPBDS) (75–77) and the McGill Pain Questionnaire (78). (Table 1 and Supplementary Table 2 (Table S2))

#### Pain interference

Eleven of the included studies measured pain interference. The most commonly used outcome measures were the bodily pain domain of the SF-36 (74) and the pain interference domain of the ISCIPBDS (75–77) (Table 1 and Supplementary Table 3 (Table S3)).

#### Health related quality of life

Fourteen of the included studies assessed changes in HRQoL. The most commonly used outcome measures were the SF-36 (74) and the International Spinal Cord Injury Quality of Life Basic Dataset (ISCIQOLBDS) (79) (Table 1 and Supplementary Table 4 (Table S4)).

### Risk of bias

Table 2 summarizes the risk of bias for the included studies as assessed by the ROB-2, EPHPP and CASP. Only one study was considered as having low risk of bias, six studies were considered to be of moderate quality, and the 23 remaining studies were deemed to be at high risk of bias. Detailed breakdown by item is available in supplementary Tables 5–7 (Table S5–S7).

**Table 2:** Summary of risk of bias assessment of included studies

Study (year)	Risk of bias tool	Risk of bias
**Alcobendas-Maestro *et al.* (2012)**	ROB-2	Moderate
**Baunsgaard *et al.* (2018)**	EPHPP	High
Benson *et al.* (2015)	EPHPP	High
**Cahill *et al.* (2018)**	CASP	Moderate
Charbonneau *et al.* (2021)	CASP	Moderate
Cinar *et al.* (2020)	EPHPP	Low
Cinar *et al.* (2021)	ROB-2	High
**Cruciger *et al.* (2016)**	EPHPP	High
**Del-ama *et al.* (2014)**	EPHPP	High
**Esquenazi *et al.* (2012)**	EPHPP	High
**Gant *et al.* (2018)**	EPHPP	High
Hu *et al.* (2023)	ROB-2	High
**Juszczak *et al.* (2018)**	EPHPP	High
**Khan *et al.* (2019)**	EPHPP	High
**Khande *et al.* (2024)**	EPHPP	Moderate
**Kim *et al.* (2021)**	EPHPP	High
**Koljonen *et al.* (2021)**	EPHPP	Moderate
**Kressler *et al.* (2014)**	EPHPP	High
Labruyere *et al.* (2014)	ROB-2	High
**Martinez *et al.* (2018)**	ROB-2	High
Mazzoleni *et al.* (2017)	EPHPP	High
**Platz *et al.* (2016)**	EPHPP	High
**Sale *et al.* (2016)**	EPHPP	High
**Sale *et al.* (2018)**	EPHPP	High
**Sawada *et al.* (2021)**	EPHPP	High
**Shackleton *et al.* (2023)**	ROB-2	Moderate
**Stampacchia *et al.* (2016)**	EPHPP	High
**Tamburella *et al.* (2020)**	EPHPP	High
Van **dijsseldonk *et al.* (2020)**	EPHPP	High
Van nes *et al.* (2022)	EPHPP	High
Van **Silfhout *et al.* (2020)**	EPHPP	High
yildiz *et al.* (2024)	EPHPP	High

Abbreviations: EPHPP, Effective Public Health Practice Project; ROB-2, Cochrane Risk of Bias Tool 2.0; CASP, Critical Appraisal Skills Programme Tool

### Meta-analysis

#### Pain intensity

**Figure 3:**
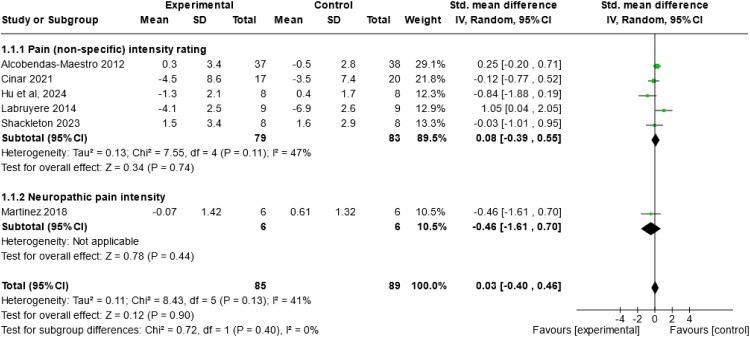
Forest plot of comparison: 1. RAGT versus comparators, outcome: Pain Intensity Rating. Values are presented as mean difference between pre- and post-intervention

Figure 3 depicts the forest plot for the outcome pain intensity with the overall result and sub-analysis presented as described below.

All phenotypes: Data from the six RCTs (48,51,56,60,63,66) (n = 174) were pooled for the primary outcome of pain intensity (all pain phenotypes considered) (Figure 3). No significant effect was found in favor of RAGT when compared to comparator interventions in reducing overall pain intensity (SMD: 0.03; 95% CI: −0.40; 0.46; Z = 0.12; P = 0.90) with moderate statistical heterogeneity (i^2 ^=  41%) observed. Comparator interventions were conventional overground walking + standard physical treatment program (56); conventional therapy (ROM/stretching exercises, strength training, standard walking) (60); combined exercise program (resistance training, cardiovascular training, flexibility exercises, gait re-education without treadmill or robotic assistance) (51); lower limb strength training (66) and routine exercise therapy (63).

Non-specified pain intensity: Data from five RCTs (n = 162) reported pain intensity where the pain phenotype was not specified. No significant effect was found in favor of RAGT compared to comparator interventions in reducing non-specified pain intensity (SMD: 0.08; 95% CI: −0.39. 0.55; Z = 0.33; P = 0.74) with low statistical heterogeneity (i^2 ^=  47%) observed. Comparator interventions were conventional overground walking + standard physical treatment program (56); conventional therapy (ROM/stretching exercises, strength training, standard walking) (60); combined exercise program (resistance training, cardiovascular training, flexibility exercises, gait re-education without treadmill or robotic assistance) (51); lower limb strength training (66) and routine exercise therapy (63).

Neuropathic pain (NP) intensity: Data from one RCT with 12 participants (48) is presented for RAGT in comparison to a multimodal exercise program for the outcome of NP intensity. While a reduction in pain intensity was observed, it did not reach statistical significance (SMD: −0.46; 95% CI: −1.61, 0.70; Z = 0.78; P = 0.44).

#### Pain interference

**Figure 4:**
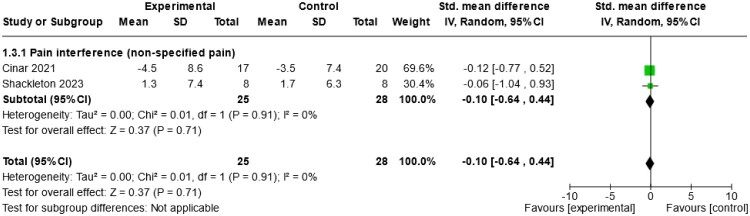
Forest plot of comparison: 2. RAGT versus comparator interventions, outcome: Pain Interference. Values are presented as mean difference between pre- and post-intervention

Figure 4 depicts the meta-analysis conducted for the outcome of pain interference. Data were pooled from two RCTs (n = 53) with a nonsignificant reduction in pain interference observed following RAGT in comparison to conventional therapy (60) and combined exercise program (51) (SMD: −0.10; 95% CI: −0.64, 0.44; Z = 0.37; P = 0.71; low statistical heterogeneity (i^2^ = 0%)).

#### Health related quality of life (HRQoL)

**Figure 5:**
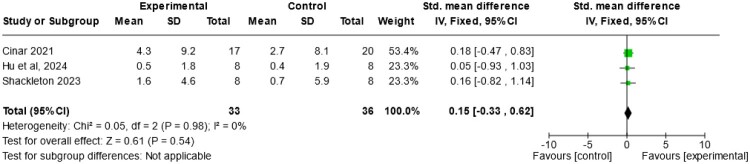
Forest plot of comparison: 3. RAGT versus comparator interventions, outcome: Health Related Quality of Life. Values are presented as mean difference between pre- and post-intervention

Figure 5 depicts the meta-analysis of RAGT in comparison to conventional therapy (60); routine exercise therapy (63) and combined exercise program (51) for the outcome of HRQoL. Three RCTs (n = 69) were pooled with a non-significant improvement observed SMD: 0.15; 95% CI: −0.33, 0.62; Z = 0.61; P = 0.54; low statistical heterogeneity (i^2 ^=  0%).

### Certainty of evidence

Table 3 summarizes overall certainty of evidence (COE) provided by the meta-analyses conducted using the GRADE approach. Very low COE was found for the results presented for the outcomes of overall pain intensity (6 RCTs (n = 174)), non-specified pain intensity (5 RCTs (n = 162)), NP intensity (1 RCT (n = 12)) and pain interference based on data from 2 RCTs (n = 53). Low COE was found for outcome HRQOL (3 RCTs (n = 69)) (48,51,56,60,63,66).

**Table 3:** Grade certainty of evidence table

Certainty assessment							Summary of findings					Importance (1–3: low importance; 4–6: important but not critical; 7–9: critical)
							No. of patients		Effect		Certainty	
No. of Studies	Study design	Risk of bias	Inconsistency	Indirectness		Imprecision	Other considerations	RAGT	Comparators	Relative (95% CI)			Absolute (95% CI)
Non-specified pain intensity												
5	RCT	Very serious ^a^	Serious ^b^	Serious ^c,d^	Serious ^e,f^	None	79	83	–	SMD 0.08 SD higher (0.39 lower to 0.55 higher)	⨁◯◯◯ Very low	6
Neuropathic pain intensity												
1	RCT	Very serious ^g^	Not serious	Not serious	Serious ^e^	None	6	6	–	SMD −0.46 SD lower (1.61 lower to 0.7 higher)	⨁◯◯◯ Very low	8
Overall pain intensity												
6	RCT	Very serious ^a^	Serious ^b^	Serious ^c,d^	Serious ^e,f^	None	85	89	–	SMD 0.03 SD higher (0.4 lower to 0.46 higher)	⨁◯◯◯ Very low	7
Health related quality of life												
3	RCT	Serious ^h^	Not serious	Not serious	Serious ^e^	None	33	36	–	SMD 0.15 SD higher (0.33 lower to 0.62 higher)	⨁⨁◯◯ Low	7
Pain interference												
2	RCT	Serious ^i^	Not serious	Serious ^j^	Serious ^e^	None	25	28	–	SMD 0.1 SD lower (0.64 lower to 0.44 higher)	⨁◯◯◯ Very low	7

Abbreviations: RCT, Randomized Controlled Trial; CI, Confidence Interval; SD, Standard Deviation; SMD, Standardized Mean Difference.

^a^ Multiple studies with high risk of bias.

^b^ Studies crossed both sides of line of zero.

^c^ Pain intensity assessed as secondary outcome.

^d^ Valid tools not used to assess pain intensity.

^e^ Wide confidence interval.

^f^ Moderate statistical heterogeneity.

^g^ High risk of bias.

^h^ Two studies with moderate risk of bias.

^i^ Study with moderate risk of bias.

^j^ One study did not directly assess pain interference.

### Narrative synthesis

#### Neuropathic pain (NP) intensity

Of the nine studies that assessed and reported neuropathic pain intensity, four studies showed positive effects for RAGT in reduction of NP intensity. Of these four studies, two contained participants with moderate-to-severe NP at baseline. One case study with two participants showed a 5.9-point reduction in NRS (0-10) from pre to post intervention, a clinically important difference (Pre: 6.5 ± 2.1; Post: 0.6 ± 0.1) (40). A case series with three participants, demonstrated a clinically meaningful effect for RAGT in affecting reductions in NP intensity in two out of the three participants (Range: 2–5 points reduction on a 0–10 scale) (41). Two qualitative studies reported subjectively reported reductions in neuropathic pain intensity following RAGT (46,47).

#### Non-specified pain-type intensity

Of the 26 studies that assessed pain intensity for a non-specified pain phenotype, six showed a significant or clinically important effect in favor of RAGT for the reduction of non-specified pain intensity (49,52,57,58,70). A pilot cohort study by Van Silfhout *et al.*, (70) showed a 1-point reduction in VAS from pre to post intervention (Pre: 2.5 ± 1.9; Post: 1.5 ± 1.8; P = 0.012). Stampacchia *et al.*, (52) in a case series of one exoskeleton training session demonstrated a clinically important mean reduction of 3.9 in NRS (0-10) from pre to post intervention in participants who reported pain at baseline, (Pre: 5.9 ± 0.6; Post: 2 ± 1.1). Khande *et al*. (49) showed a significant improvement in VAS score from pre to post intervention in both RAGT and comparator groups (who received conventional physiotherapy) (RAGT: Pre: 7.2 ± 0.6; Post: 5.3 ± 0.6; P = 0.001; Comparator: Pre: 7.3 ± 0.6; Post: 5.7 ± 0.6; P = 0.001) but no between group difference (P = 0.099). A cohort study by Yildiz *et al.* (71) showed a significant improvement in the physical health domain of the (Pre: 41.9 ± 10.2; Post: 49.7 ± 6.8; P < 0.001). Two qualitative studies reported reductions in pain following RAGT (57,58). The remaining 22 studies showed no effect in favor of RAGT for reducing non-specified pain intensity.

#### Pain interference

Of the eleven studies that assessed pain interference, only one case study who recruited participants with moderate-to-severe NP (40) demonstrated a clinically meaningful improvement following RAGT.

#### Health related quality of life

Of the 14 studies that assessed HRQoL, one case study who recruited participants with moderate-to-severe NP (40) and one prospective comparative study with participants with severe non-specified pain intensity at baseline (49) demonstrated clinically meaningful or significant improvements following RAGT. One cohort study (71), found significant improvements in all domains of the WHOQOL-BREF. In addition, Shackleton and colleagues (51) found significant improvements in the physical domain of the ISCIQOLBDS in favor of RAGT compared to a combined exercise program but no differences were observed for general and psychological domains or overall scores.

### Best evidence synthesis

Table 4 presents a best evidence synthesis according to Sackett’s level of evidence (1989, 38) for the efficacy of RAGT across all outcomes of interest.

**Table 4:** Best evidence synthesis statement

Research questions	Best evidence synthesis statement
1. What evidence currently supports RAGT as a treatment for those with neuropathic pain after SCI to reduce pain intensity levels?	Level IV evidence from two CAS/CSS, with high ROB demonstrates clinically meaningful changes in neuropathic pain intensity after 6–12 weeks of RAGT in individuals with moderate-to-severe baseline neuropathic pain intensity.
2. What evidence currently supports RAGT to improve neuropathic pain interference following SCI?	Level IV evidence from one case study, with high ROB demonstrates clinically significant improvements in neuropathic pain interference after 12 weeks of RAGT for those with severe neuropathic pain interference at baseline.
3. What evidence currently supports RAGT to improve health-related quality of life for people with neuropathic pain following SCI?	Level IV evidence from one case study, with high ROB demonstrates significant improvement in health-related quality of life after 12 weeks of RAGT for those with moderate-to-severe neuropathic pain at baseline.

Abbreviations: CSS, Case Series; CAS, Case Study; ROB: Risk of Bias.

## Discussion

This systematic review collated all evidence from studies that included a measure of pain during RAGT after SCI. Based on the currently published evidence, there are no conclusive findings to be drawn from RCTs assessing pain or NP reduction following RAGT, acknowledging that none of these studies explicitly recruited participants with NP at baseline. Indeed, it is notable that from the 32 studies included in this review, only 2 case series specifically recruited people with NP at baseline (40,41). In both of these studies, clinically significant reductions in NP intensity were observed. Meta-analyses conducted in this review for the outcome of pain intensity, including one study recording NP pain intensity demonstrated non-significant effects in a SCI population that were heterogeneous in their pain profiles. This lack of conclusive evidence is disappointing given the economic burden of NP in SCI patients and their high healthcare utilization (80) and has implications when seeking to justify funding of RAGT treatments (81).

There was heterogeneity in terms of the RAGT modalities used (over treadmill, overground, end-effector, etc) as well as the study settings. Although differences have been identified between different types of RAGT devices in domains such as trunk muscle activation (82), there is inconclusive evidence that RAGT modality or study setting have any effect on NP intensity, interference or HRQoL. These findings mirrors that of previous work that has not been able to establish whether one modality is superior to another, primarily due to lack of studies comparing the effects of different types of RAGT modality. A recent systematic review found that no studies currently exist comparing two or more different types of RAGT modalities across 14 different domains of interest including HRQoL (83).

It is evident from the findings of this review that the majority of studies of RAGT in SCI identified were designed to examine efficacy for gait-related outcomes. This is a well-studied area with a number of published systematic reviews available (23,24,25). As such, pain intensity was assessed as a secondary outcome measure and with the exception of one case study (40), participants were not required to have pain or NP at baseline for study entry. Subsequently studies presented with mixed populations (pain and no pain) with many participants presenting with low pain intensity levels or no pain at baseline (Table 1). Only seven studies contained participants with moderate-to-severe pain at baseline with only two of these studies reporting the pain type as neuropathic pain. The low pain intensity levels reported in many of the studies in this review does not accurately reflect the contribution of pain to disease burden and reduced quality of life post-SCI (6,7). Despite numerous studies showing that people with SCI prioritize addressing secondary complications including pain (and their subsequent effects on HRQoL and their participation in their communities) even over improvement in gait function (84–86), this review highlights an inadequate focus on pain in SCI research, particularly in the context of RAGT.

Currently only one RCT with a crossover design was identified that analysed the effects of RAGT on what they reported as NP intensity (48). It is interesting to note, in the context of this review, that the RCT did not target participants with moderate-to-severe NP at baseline and had an active comparator intervention that comprised a multimodal exercise program providing sensorimotor stimulation. These factors may well impact the NP outcomes in both groups, as emerging evidence supports sensorimotor activity as a beneficial approach for modulating NP in both animals and humans (19–21). In addition, it has been posited that walking may be more efficacious than other exercise modalities such as swimming and other RAGT activities such as transfer practice and standing alone, due to the characteristics of rhythmic stimulation of proprioceptive and mechanosensory afferent inputs in conjunction with weight bearing (21).

Level IV evidence from two case studies/series supports the use of RAGT in the amelioration of NP intensity (40,41). Despite the lower level of evidence provided by these case studies, they were better placed to examine RAGT on NP intensity than the RCT by Martinez and colleagues (48) owing to the fact that moderate-to-severe NP at baseline was an inclusion criterion and confounders in a comparator group were absent. In fact both these studies reported improvements above the MCID for pain intensity, considered as 30% or 1.86-point reduction in scores (45,87) and mirror findings from studies assessing virtual walking for NP after SCI (88). Well conceptualized RCTs are required that recruit individuals with established and moderate-to-severe NP post-SCI and that compare the RAGT intervention with a control that is devoid of sensorimotor stimulation to establish the mechanistic basis for RAGT as a treatment for NP.

Of the eleven total studies included this review that assessed changes in pain interference, only the two case studies/series identified in the literature assessed it in the context of NP (40,41). Similarly, out of the 13 studies that assessed changes in HRQoL, just one case study assessed this in the context of NP (40). This is disappointing given, NP in people living with SCI interferes with HRQoL domains including sleep and mood, as well as impacting overall HRQoL significantly more than other pain phenotypes (6,7). In addition, only four studies that assessed pain interference and two studies that assessed HRQoL across the full review used outcome measures that have been validated in a SCI population such as the ISCIPBDS (75–77) and the ISCIQOLBDS (79). This problem extended to the assessment of pain intensity also, with just four studies using tools validated in a SCI population.

A key issue identified in the majority of the literature in this review was the failure to adequately define or classify participants’ pain based on phenotype. We reported the pain intensity outcomes from these studies as non-specified pain. Ambiguity surrounding pain may also lead to over-estimation of non-specified pain within this population (2). However, the participants in these studies could have presented with NP, nociceptive pain and/or visceral pain that was not clearly identified. This is especially important in cases where pain intensity was wrongly classified as NP or where pain, considered in general terms related to musculoskeletal or visceral problems. In all of these cases, RAGT as a sensorimotor stimulation intervention to reverse the maladaptive central changes eliciting NP, would not have mechanistic basis for amelioration of pain (19). Other studies such as Cruciger *et al.*, (40) targeted participants with NP, but did not thoroughly describe how they classified or diagnosed their participants’ pain as NP, a common methodological issue across the literature (2).

### Limitations

This review and its findings must be interpreted in light of a number of considerations and limitations. Studies published in languages other than English were excluded and it is possible that relevant studies in other languages may have been missed and thus were not included in this review. This was primarily due to limited time and monetary resources. An update of this review would benefit from international collaboration to try to address the language bias. Many of the included studies in the narrative review and best evidence synthesis addressing NP did not report P-values or effect sizes, had small sample sizes and were deemed to be at a high risk of bias. As a result, cautious interpretation of these results is required. The certainty of evidence from the available RCTs (Table 2) for outcomes pain intensity, pain interference and HRQoL following RAGT was deemed low to very low, meaning there can be little confidence in their true effect. Furthermore, the review, based on currently published evidence, was unable to answer, using high level meta-analysis, the question of whether RAGT reduces NP intensity or interference.

The included studies which did report NP rarely diagnosed it in line with best level evidence (89). Without using rigorous screening and diagnostic standard criteria to accurately identify NP, participants may present with nociceptive, visceral, or mixed phenotype pain that would not be expected to change after RAGT where neurophysiological mechanisms of central NP relief contrast from those of other pain phenotypes.

### Recommendations

To examine the true effect of RAGT, as a mechanistic treatment for SCI generated NP, we recommend that future research target participants with moderate to severe pain at baseline (*i.e.* at least 4/10 or 6/10 respectively on the NRS) and with NP phenotype that has been accurately classified and diagnosed in line with best level evidence guidelines at the recruitment phase of the trial (2,89). With no gold-standard for diagnosing NP, the IASP Neuropathic Pain Special Interest Group (NeuPSIG) (89) nonetheless provides strong recommendations to use a validated screening questionnaires with high sensitivity and specificity such as The Douleur Neuropathique 4 (DN4) (90). In addition, for a more accurate estimate of treatment effects, future studies should use tools validated in a SCI population to assess changes in NP intensity and pain interference such as the ISCIPBDS (75–77), NP symptom severity such as the Neuropathic Pain Symptom Inventory (NPSI) (91,92) and HRQoL such as the ISCIQOLBDS (79). A recently published protocol for a randomized feasibility trial (93) is looking to target these current gaps in the literature which is a positive development in this research area.

## Conclusion

Limited and low-quality evidence currently supports the use of RAGT in the reduction of SCI NP, primarily related to a lack of studies focussed on RAGT as a NP treatment. Well conceptualized RCTs are required that recruit individuals with established, accurately diagnosed moderate-to-severe neuropathic pain post SCI and that compare RAGT with a control that is devoid of motor stimulation to establish the mechanistic basis for RAGT as a treatment for NP.

## Supplementary Material

List and description of supplementary materials JSCM.docx

Supplementary Materials SR 2 updated.docx
