# Supplementary material for: Robotic-assisted gait training for spinal cord injury neuropathic pain: A systematic review
Source: J Spinal Cord Med. 2025 Jun 6;49(4):635–61. doi: 10.1080/10790268.2025.2503049 (PMC13295081; doi:10.1080/10790268.2025.2503049)
Supplement: List and description of supplementary materials JSCM.docx [file YSCM_A_2503049_SM1908.docx]

**List and description of supplementary materials:**

**Table S1:** Outcome Measures used in studies assessing non-specified pain intensity

**Table S2:** Outcome Measures used in studies assessing neuropathic pain intensity

**Table S3:** Outcome measures used in studies assessing pain interference

**Table S4:** Outcome measures used in studies assessing health related quality of life

**Table S5:** Risk of Bias for RCTs (ROB 2)

**Table S6:** Risk of Bias for non-RCT quantitative studies (EPHPP)

**Table S7:** Risk of bias for qualitative studies (CASP)

**Table S8:** Search Strategy

**Table S9:** PRISMA Abstract Checklist for Systematic Reviews

**Table S10:** PRISMA Checklist for Systematic Reviews
