# Supplementary material for: Robotic-assisted gait training for spinal cord injury neuropathic pain: A systematic review
Source: J Spinal Cord Med. 2025 Jun 6;49(4):635–61. doi: 10.1080/10790268.2025.2503049 (PMC13295081; doi:10.1080/10790268.2025.2503049)
Supplement: Supplementary Materials SR 2 updated.docx [file YSCM_A_2503049_SM1907.docx]

Table S1: Outcome Measures used in studies assessing non-specified pain intensity:

| **Study Author** | **Outcome Measure** | **Notes** |
| --- | --- | --- |
| Van Silfhout et al. (2020) | VAS | N/A |
| Sale et al. (2018) | VAS | N/A |
| Esquenazi et al. (2012) | VAS | N/A |
| Del-Ama et al. (2014) | VAS | N/A |
| Benson et al. (2016) | VAS | N/A |
| Labruyere et al. (2014) | VAS | N/A |
| Alcobendas-Maestro et al. (2012) | VAS | N/A |
| Tamburella et al. (2020) | VAS | N/A |
| Sale et al. (2016) | VAS | N/A |
| Khande et al. (2024) | VAS | N/A |
| Stampacchia et al. (2016) | NRS | N/A |
| Mazzoleni et al. (2017) | NRS | N/A |
| Koljonen et al. (2021) | NRS | N/A |
| Khan et al. (2019) | NRS | N/A |
| Juszczak et al. (2018) | NRS | N/A |
| Van Nes et al. (2022) | SF-36 | Bodily-pain domain |
| Sawada et al. (2021) | SF-36 | Bodily-pain domain |
| Kim et al. (2021) | SF-36 | Bodily-pain domain |
| Cinar et al. (2020) | SF-36 | Bodily-pain domain |
| Cinar et al. (2021) | SF-36 | Bodily-pain domain |
| Platz et al. (2012) | SF-12 | Bodily-pain domain |
| Hu et al. (2023) | WHOQOL-BREF | Physical health subdomain |
| Yildiz et al. (2024) | WHOQOL-BREF | Physical health subdomain |
| Cahill et al. (2018) | Qualitative reports | N/A |
| Charbonneau et al. (2021) | Qualitative reports | N/A |
| Shackleton et al. (2023) | ISCIPBDS | Pain intensity and location in previous week |

**Abbreviations:** ISCIPBDS, International Spinal Cord Injury Pain Basic Dataset; NRS, Numerical Rating Scale; SF-12, 12 item Short Form Survey; SF-36, 36-Item Short Form Survey; VAS, Visual Analog Scale; WHOQOL-BREF, World Health Organization Quality of Life- Abbreviated Version;

Table S2: Outcome Measures used in studies assessing neuropathic pain intensity:

| **Study Author** | **Outcome Measure** | **Notes** |
| --- | --- | --- |
| Kressler et al. (2014) | ISCIPBDS | N/A |
| Baunsgaard et al. (2018) | ISCIPBDS | Pain location. Result expressed as percentage of participants who experienced pain a week before intervention and during the intervention. |
| Gant et al. (2018) | ISCIPBDS | Pain location, intensity and type in past week. |
| Khan et al. (2019) | MPQ | N/A |
| Martinez et al. (2018) | MPQ | N/A |
| Van Dijsseldonk et al. (2020) | Qualitative reports | N/A |
| Platz et al. (2016) | Qualitative reports | N/A |
| Sawada et al. (2021) | NPSI | N/A |
| Cruciger et al. (2016) | NRS | N/A |

**Abbreviations:** ISCIPBDS, International Spinal Cord Injury Pain Basic Dataset; NPSI, Neuropathic Pain Symptom Inventory; NRS, Numerical Rating Scale; MPQ, McGill Pain Questionnaire

Table S3: Outcome measures used in studies assessing pain interference:

| **Study Author** | **Outcome Measure** | **Notes** |
| --- | --- | --- |
| Cinar et al. (2020) | SF-36 | Bodily-pain domain |
| Van Nes et al. (2022) | SF-36 | Bodily-pain domain |
| Sawada et al. (2021) | SF-36 | Bodily-pain domain |
| Kim et al. (2021) | SF-36 | Bodily-pain domain |
| Cinar et al. (2021) | SF-36 | Bodily-pain domain |
| Cruciger et al. (2016) | SF-36 | Bodily-pain domain |
| Gant et al. (2018) | ISCIPBDS | Pain interference with sleep |
| Baunsgaard et al. (2018) | ISCIPBDS | Pain interference with mood, daily activities and sleep. |
| Shackleton et al. (2018) | ISCIPBDS | Pain interference with mood, daily activities and sleep. |
| Kressler et al. (2014) | ISCIPBDS | Pain interference with sleep |
| Platz et al. (2016) | SF-12 | Bodily-pain domain |

**Abbreviations:** ISCIPBDS, International Spinal Cord Injury Pain Basic Dataset; SF-12, 12 item Short Form Survey; SF-36, 36-Item Short Form Survey;

Table S4: Outcome measures used in studies assessing health related quality of life:

| **Study Author** | **Outcome Measure** | **Notes** |
| --- | --- | --- |
| Cinar et al. (2020) | SF-36 | N/A |
| Van Nes et al. (2022) | SF-36 | N/A |
| Sawada et al. (2021) | SF-36 | N/A |
| Kim et al. (2021) | SF-36 | N/A |
| Cinar et al. (2021) | SF-36 | N/A |
| Cruciger et al. (2016) | SF-36 | N/A |
| Shackleton et al. (2023) | ISCIQOLBDS | N/A |
| Mazzoleni et al. (2017) | ISCIQOLBDS | N/A |
| Baunsgaard et al. (2018) | ISCIQOLBDS | N/A |
| Yildiz et al. (2024) | WHOQOL-BREF | N/A |
| Hu et al. (2023) | WHOQOL-BREF | N/A |
| Benson et al. (2016) | ATD PA | N/A |
| Juszczak et al. (2018) | SWLS | N/A |
| Khande et al. (2024) | MGQOL | N/A |
| Platz et al. (2016) | SF-12 | N/A |

**Abbreviations:** ATD-PA, Assistive Technology Device Predisposition Assessment; ISCIQOLBDS, International Spinal Cord Injury Quality of Life Basic Dataset; MGQOL, McGill Quality of Life Questionnaire; SF-12, 12 item Short Form Survey; SF-36, 36-Item Short Form Survey; SWLS, Satisfaction with Life Scale; WHOQOL-BREF, World Health Organization Quality of Life- Abbreviated Version

Table S5: Risk of Bias for RCTs (ROB 2):

| **STUDY**  **(YEAR)** | D1: Randomisation Process | DS: Period and Carryover effects | D2: Deviations from intended interventions | D3: Missing outcome data | D4: Measurement of outcome | D5: Selection of reported result | **OVERALL QUALITY** |
| --- | --- | --- | --- | --- | --- | --- | --- |
| Alcobendas-Maestro et al. (2012) | ++ | N/A | + | ++ | ++ | ++ | + |
| Cinar et al.  (2021) | ++ | N/A | + | ++ | - | ++ | - |
| Hu et al.  (2023) | ++ | N/A | + | ++ | - | ++ | - |
| Labruyere and Van Hedel  (2014) | + | - | + | ++ | ++ | ++ | - |
| Martinez et al.  (2018) | ++ | ++ | + | - | ++ | ++ | - |
| Shackleton et al.  (2023) | ++ | N/A | + | ++ | ++ | ++ | + |

| -  HIGH | +  SOME CONCERNS | ++  LOW | N/A |
| --- | --- | --- | --- |

Table S6: Risk of Bias for non-RCT quantitative studies (EPHPP):

| **STUDY**  **(YEAR)** | A: Selection Bias | B: Study Design | C: Confounders | D: Blinding | E: Data Collection Methods | F: Withdrawals and Dropouts | **OVERALL QUALITY** |
| --- | --- | --- | --- | --- | --- | --- | --- |
| Baunsgaard et al.  (2018) | + | + | - | - | ++ | ++ | - |
| Benson et al.  (2015) | - | + | - | - | ++ | - | - |
| Cinar et al.  (2020) | + | + | ++ | + | ++ | ++ | ++ |
| Cruciger et al.  (2016) | - | - | - | - | ++ | - | - |
| Del-Ama et al.  (2014) | - | + | - | - | ++ | - | - |
| Esquenazi et al.  (2012) | - | + | - | - | ++ | ++ | - |
| Gant et al.  (2018) | - | + | - | - | - | ++ | - |
| Juszczak et al.  (2018) | + | + | - | - | ++ | - | - |
| Khan et al.  (2019) | - | + | - | - | ++ | ++ | - |
| Khande et al.  (2024) | + | + | ++ | - | ++ | ++ | + |
| Kim et al.  (2021) | - | + | - | - | ++ | ++ | - |
| Koljonen et al.  (2021) | + | + | ++ | - | ++ | ++ | + |
| Kressler et al.  (2014) | - | - | - | - | ++ | - | - |
| Mazzoleni et al.  (2017) | - | + | - | - | ++ | ++ | - |
| Platz et al.  (2016) | - | + | - | - | ++ | ++ | - |
| Sale et al.  (2016) | - | - | - | + | - | ++ | - |
| Sale et al.  (2018) | - | + | - | - | ++ | ++ | - |
| Sawada et al.  (2021) | - | + | - | - | ++ | - | - |
| Stampacchia et al.  (2016) | - | + | - | - | ++ | - | - |
| Tamburella et al.  (2020) | - | + | - | - | ++ | ++ | - |
| Van Dijsseldonk et al.  (2020) | - | - | - | - | - | - | - |
| Van Nes et al.  (2022) | - | + | - | - | ++ | ++ | - |
| Van Silfhout et al.  (2020) | - | + | - | - | ++ | - | - |
| Yildiz et al.  (2024) | - | + | - | - | ++ | - | - |

| -  WEAK | + MODERATE | ++ STRONG |
| --- | --- | --- |

Table S7: Risk of bias for qualitative studies (CASP):

| **STUDY**  **(YEAR)** | Q1 | Q2 | Q3 | Q4 | Q5 | Q6 | Q7 | Q8 | Q9 | Q10 | **OVERALL**  **QUALITY** |
| --- | --- | --- | --- | --- | --- | --- | --- | --- | --- | --- | --- |
| Cahill et al.  (2018) | Y | Y | Y | N | CT | CT | Y | Y | Y | Y | **+** |
| Charbonneau et al.  (2021) | Y | Y | Y | CT | Y | CT | CT | Y | Y | Y | **+** |

**Abbreviations:** CT, Can’t Tell; N, No, Y, Yes.

| -  WEAK | + MODERATE | ++ YES/STRONG |
| --- | --- | --- |

Table S8: Search Strategy

| **Database** | **Spinal Cord Injury** | **Robotic-Assisted Gait Device** |
| --- | --- | --- |
| **Pubmed** | "spinal cord" **AND** (wound* OR trauma OR injur* OR damag* or lesion* OR contusion* OR laceration* OR isch* OR disease* OR dysfunction)  **OR**  "Spinal cord injur*" OR Parapleg* OR Quadripleg* OR Parapare* OR Quadripare* OR Tetrapleg* OR Tetrapare* OR "Central cord syndrome" OR "Central spinal cord syndrome" OR “Central cord injury syndrome” OR "Spinal tumour*" OR “Spinal tumor*” OR "Syringomyelia" OR "Spinal stroke" OR "Spinal cord dysfunction" OR Spinal cord injuries[MeSH] OR Paraplegia[MeSH] OR Quadriplegia[MeSH] | ("robotic assisted" OR "robotic-assisted" OR "orthotic" OR "exoskeleton" OR "electromechanical-assisted" OR "electromechanical assisted" OR "electromechanical" OR "electro-mechanical" OR mechanized OR mechanised OR powered OR robot* OR "powered robotic") **AND** (gait OR locomot* OR device* OR machine OR exoskeleton OR train* OR therap* OR exercise OR rehabilitat* or re-educat*)  **OR**  "Hybrid assistive limb" OR "Driven gait orthosis" OR "Powered gait orthosis" OR "end-effector device*" OR Robot* OR Automat* OR "Man-Machine System*" OR "Self-Help Device*" OR "G-Eo System" OR "Lokohelp" OR "LoPES" OR "Lokomat" OR "Ekso Bionics" OR "REX P" OR ReWalk OR "Kinetron" OR "Anklebot" OR "AutoAmbulator" OR "Locomat" OR "Exoskeleton device"[MeSH] OR "Orthotic devices"[MeSH] OR Robotics[MeSh] OR Automation[MeSh] OR "Man-Machine Systems"[MeSH] OR "Self-Help Devices"[MeSH] |
| **Embase** | ‘spinal cord’ **AND** (wound* OR trauma OR injur* OR damag* or lesion* OR contusion* OR laceration* OR isch* OR disease* OR dysfunction)  **OR**  ‘Spinal cord injur*’ OR Parapleg* OR Quadripleg* OR Parapare* OR Quadripare* OR Tetrapleg* OR Tetrapare* OR ‘Central cord syndrome’ OR ‘Central spinal cord syndrome’ OR ‘Central cord injury syndrome’ OR ‘Spinal tumour*’ OR ‘Spinal tumor*’ OR ‘Syringomyelia’ OR ‘Spinal stroke’ OR ‘Spinal cord dysfunction’ OR ‘spinal cord injury’/exp OR ‘paraplegia’/exp OR ‘quadriplegia’/exp | (‘robotic assisted’ OR ‘robotic-assisted’ OR ‘orthotic’ OR ‘exoskeleton’ OR ‘electromechanical-assisted’ OR ‘electromechanical assisted’ OR ‘electromechanical’ OR ‘electro-mechanical’ OR mechanized OR mechanised OR powered OR robot* OR ‘powered robotic’) **AND** (gait OR locomot* OR device* OR machine OR exoskeleton OR train* OR therap* OR exercise OR rehabilitat* or re-educat*)  **OR**  ‘Hybrid assistive limb’ OR ‘Driven gait orthosis’ OR ‘Powered gait orthosis’ OR ‘end-effector device*’ OR Robot* OR Automat* OR ‘Man-Machine System*’ OR ‘Self-Help Device*’ OR ‘G-Eo System’ OR ‘Lokohelp’ OR ‘LoPES’ OR ‘Lokomat’ OR ‘Ekso Bionics’ OR ‘REX P’ OR ReWalk OR ‘Kinetron’ OR ‘Anklebot’ OR ‘AutoAmbulator’ OR ‘Locomat’ OR 'exoskeleton (rehabilitation)'/exp OR 'orthosis'/exp OR 'automation'/exp OR 'self help device'/exp OR 'man machine interaction'/exp |
| **CINAHL** | TX ( "spinal cord" **AND** (wound* OR trauma OR injur* OR damag* or lesion* OR contusion* OR laceration* OR isch* OR disease* OR dysfunction) )  **OR**  TX ( "spinal cord injur*" OR parapleg* OR quadripleg* OR parapare* OR quadripare* OR tetrapleg* OR tetrapare* OR "Central cord syndrome" OR "Central spinal cord syndrome" OR “Central cord injury syndrome” OR "Spinal tumour*" OR “Spinal tumor*” OR "Syringomyelia" OR "Spinal stroke" OR "Spinal cord dysfunction" ) OR TX ( (MH "Spinal Cord Injuries+") OR (MH "Paraplegia+") OR (MH "Quadriplegia+") ) | TX ( ("robotic assisted" OR "robotic-assisted" OR "orthotic" OR "exoskeleton" OR "electromechanical-assisted" OR "electromechanical assisted" OR "electromechanical" OR "electro-mechanical" OR mechanized OR mechanised OR powered OR robot* OR "powered robotic") **AND** (gait OR locomot* OR device* OR machine OR exoskeleton OR train* OR therap* OR exercise OR rehabilitat* or re-educat*) )  **OR**  TX ( "Hybrid assistive limb" OR "Driven gait orthosis" OR "Powered gait orthosis" OR "end-effector device*" OR Robot* OR Automat* OR "Man-Machine System*" OR "Self-Help Device*" OR "G-Eo System" OR "Lokohelp" OR "LoPES" OR "Lokomat" OR "Ekso Bionics" OR "REX P" OR ReWalk OR "Kinetron" OR "Anklebot" OR "AutoAmbulator" OR "Locomat" ) OR TX ( (MH "Exoskeleton Devices") OR (MH "Orthoses+") OR (MH "Robotics+") OR (MH "Automation+") OR (MH "Assistive Technology Devices+") ) |
| **SCOPUS** | "spinal cord" AND (wound* OR trauma OR injur* OR damag* or lesion* OR contusion* OR laceration* OR isch* OR disease* OR dysfunction)  **OR**  "Spinal cord injur*" OR Parapleg* OR Quadripleg* OR Parapare* OR Quadripare* OR Tetrapleg* OR Tetrapare* OR "Central cord syndrome" OR "Central spinal cord syndrome" OR “Central cord injury syndrome” OR "Spinal tumour*" OR “Spinal tumor*” OR "Syringomyelia" OR "Spinal stroke" OR "Spinal cord dysfunction" | "robotic assisted" OR "robotic-assisted" OR "orthotic" OR "exoskeleton" OR "electromechanical-assisted" OR "electromechanical assisted" OR "electromechanical" OR "electro-mechanical" OR mechanized OR mechanised OR powered OR robot* OR "powered robotic" **AND** gait OR locomot* OR device* OR machine OR exoskeleton OR train* OR therap* OR exercise OR rehabilitat* or re-educat*  **OR**  "Hybrid assistive limb" OR "Driven gait orthosis" OR "Powered gait orthosis" OR "end-effector device*" OR Robot* OR Automat* OR "Man-Machine System*" OR "Self-Help Device*" OR "G-Eo System" OR "Lokohelp" OR "LoPES" OR "Lokomat" OR "Ekso Bionics" OR "REX P" OR ReWalk OR "Kinetron" OR "Anklebot" OR "AutoAmbulator" OR "Locomat" |
| **Web of Science** | ‘spinal cord’ AND (wound* OR trauma OR injur* OR damag* or lesion* OR contusion* OR laceration* OR isch* OR disease* OR dysfunction)  **OR**  ‘Spinal cord injur*’ OR Parapleg* OR Quadripleg* OR Parapare* OR Quadripare* OR Tetrapleg* OR Tetrapare* OR ‘Central cord syndrome’ OR ‘Central spinal cord syndrome’ OR ‘Central cord injury syndrome’ OR ‘Spinal tumour*’ OR ‘Spinal tumor*’ OR ‘Syringomyelia’ OR ‘Spinal stroke’ OR ‘Spinal cord dysfunction’ | (‘robotic assisted’ OR ‘robotic-assisted’ OR ‘orthotic’ OR ‘exoskeleton’ OR ‘electromechanical-assisted’ OR ‘electromechanical assisted’ OR ‘electromechanical’ OR ‘electro-mechanical’ OR mechanized OR mechanised OR powered OR robot* OR ‘powered robotic’) **AND** (gait OR locomot* OR device* OR machine OR exoskeleton OR train* OR therap* OR exercise OR rehabilitat* or re-educat*)  **OR**  ‘Hybrid assistive limb’ OR ‘Driven gait orthosis’ OR ‘Powered gait orthosis’ OR ‘end-effector device*’ OR Robot* OR Automat* OR ‘Man-Machine System*’ OR ‘Self-Help Device*’ OR ‘G-Eo System’ OR ‘Lokohelp’ OR ‘LoPES’ OR ‘Lokomat’ OR ‘Ekso Bionics’ OR ‘REX P’ OR ReWalk OR ‘Kinetron’ OR ‘Anklebot’ OR ‘AutoAmbulator’ OR ‘Locomat’ |
| **Cochrane Library** | "spinal cord" AND (wound* OR trauma OR injur* OR damag* or lesion* OR contusion* OR laceration* OR isch* OR disease* OR dysfunction)  **OR**  "Spinal cord injur*" OR Parapleg* OR Quadripleg* OR Parapare* OR Quadripare* OR Tetrapleg* OR Tetrapare* OR "Central cord syndrome" OR "Central spinal cord syndrome" OR “Central cord injury syndrome” OR "Spinal tumour*" OR “Spinal tumor*” OR "Syringomyelia" OR "Spinal stroke" OR "Spinal cord dysfunction" OR Spinal cord injuri(MeSH) OR Paraplegia(MeSH) OR Quadriplegia(MeSH) | ("robotic assisted" OR "robotic-assisted" OR "orthotic" OR "exoskeleton" OR "electromechanical-assisted" OR "electromechanical assisted" OR "electromechanical" OR "electro-mechanical" OR mechanized OR mechanised OR powered OR robot* OR "powered robotic") **AND** (gait OR locomot* OR device* OR machine OR exoskeleton OR train* OR therap* OR exercise OR rehabilitat* or re-educat*)  **OR**  "Hybrid assistive limb" OR "Driven gait orthosis" OR "Powered gait orthosis" OR "end-effector device*" OR Robot* OR Automat* OR "Man-Machine System*" OR "Self-Help Device*" OR "G-Eo System" OR "Lokohelp" OR "LoPES" OR "Lokomat" OR "Ekso Bionics" OR "REX P" OR ReWalk OR "Kinetron" OR "Anklebot" OR "AutoAmbulator" OR "Locomat" OR "Exoskeleton device"(MeSH) OR "Orthotic devices"(MeSH) OR Robotics(MeSH) OR Automation(MeSH) OR "Man-Machine Systems"(MeSH) OR "Self-Help Devices"(MeSH) |

| **Section and Topic** | **Item #** | **Checklist item** | **Reported (Yes/No)** |
| --- | --- | --- | --- |
| **TITLE** | | |  |
| Title | 1 | Identify the report as a systematic review. | Yes |
| **BACKGROUND** | | |  |
| Objectives | 2 | Provide an explicit statement of the main objective(s) or question(s) the review addresses. | Yes |
| **METHODS** | | |  |
| Eligibility criteria | 3 | Specify the inclusion and exclusion criteria for the review. | Yes |
| Information sources | 4 | Specify the information sources (e.g. databases, registers) used to identify studies and the date when each was last searched. | Yes |
| Risk of bias | 5 | Specify the methods used to assess risk of bias in the included studies. | Yes |
| Synthesis of results | 6 | Specify the methods used to present and synthesise results. | Yes |
| **RESULTS** | | |  |
| Included studies | 7 | Give the total number of included studies and participants and summarise relevant characteristics of studies. | Yes |
| Synthesis of results | 8 | Present results for main outcomes, preferably indicating the number of included studies and participants for each. If meta-analysis was done, report the summary estimate and confidence/credible interval. If comparing groups, indicate the direction of the effect (i.e. which group is favoured). | Yes |
| **DISCUSSION** | | |  |
| Limitations of evidence | 9 | Provide a brief summary of the limitations of the evidence included in the review (e.g. study risk of bias, inconsistency and imprecision). | Yes |
| Interpretation | 10 | Provide a general interpretation of the results and important implications. | Yes |
| **OTHER** | | |  |
| Funding | 11 | Specify the primary source of funding for the review. | Yes |
| Registration | 12 | Provide the register name and registration number. | Yes |

Table S9: PRISMA Abstract Checklist for Systematic Reviews

| **Section and Topic**  Table S10: PRISMA Checklist for Systematic Reviews | **Item #** | **Checklist item** | **Location where item is reported** |
| --- | --- | --- | --- |
| **TITLE** | | |  |
| Title | 1 | Identify the report as a systematic review. | Title |
| **ABSTRACT** | | |  |
| Abstract | 2 | See the PRISMA 2020 for Abstracts checklist. | Abstract |
| **INTRODUCTION** | | |  |
| Rationale | 3 | Describe the rationale for the review in the context of existing knowledge. | Introduction |
| Objectives | 4 | Provide an explicit statement of the objective(s) or question(s) the review addresses. | Introduction |
| **METHODS** | | |  |
| Eligibility criteria | 5 | Specify the inclusion and exclusion criteria for the review and how studies were grouped for the syntheses. | Methods: Eligibility criteria |
| Information sources | 6 | Specify all databases, registers, websites, organisations, reference lists and other sources searched or consulted to identify studies. Specify the date when each source was last searched or consulted. | Methods: Search strategy |
| Search strategy | 7 | Present the full search strategies for all databases, registers and websites, including any filters and limits used. | Supplementary Materials: Table S8 |
| Selection process | 8 | Specify the methods used to decide whether a study met the inclusion criteria of the review, including how many reviewers screened each record and each report retrieved, whether they worked independently, and if applicable, details of automation tools used in the process. | Methods: Study selection |
| Data collection process | 9 | Specify the methods used to collect data from reports, including how many reviewers collected data from each report, whether they worked independently, any processes for obtaining or confirming data from study investigators, and if applicable, details of automation tools used in the process. | Methods: data extraction |
| Data items | 10a | List and define all outcomes for which data were sought. Specify whether all results that were compatible with each outcome domain in each study were sought (e.g. for all measures, time points, analyses), and if not, the methods used to decide which results to collect. | Methods: Eligibility criteria |
|  | 10b | List and define all other variables for which data were sought (e.g. participant and intervention characteristics, funding sources). Describe any assumptions made about any missing or unclear information. | Methods: Data extraction |
| Study risk of bias assessment | 11 | Specify the methods used to assess risk of bias in the included studies, including details of the tool(s) used, how many reviewers assessed each study and whether they worked independently, and if applicable, details of automation tools used in the process. | Methods: Risk of bias assessment |
| Effect measures | 12 | Specify for each outcome the effect measure(s) (e.g. risk ratio, mean difference) used in the synthesis or presentation of results. | Methods: Synthesis of Results and analysis |
| Synthesis methods | 13a | Describe the processes used to decide which studies were eligible for each synthesis (e.g. tabulating the study intervention characteristics and comparing against the planned groups for each synthesis (item #5)). | Methods: Synthesis of Results and analysis |
|  | 13b | Describe any methods required to prepare the data for presentation or synthesis, such as handling of missing summary statistics, or data conversions. | Methods: Synthesis of Results and analysis |
|  | 13c | Describe any methods used to tabulate or visually display results of individual studies and syntheses. | Methods: Synthesis of Results and analysis |
|  | 13d | Describe any methods used to synthesize results and provide a rationale for the choice(s). If meta-analysis was performed, describe the model(s), method(s) to identify the presence and extent of statistical heterogeneity, and software package(s) used. | Methods: Synthesis of Results and analysis |
|  | 13e | Describe any methods used to explore possible causes of heterogeneity among study results (e.g. subgroup analysis, meta-regression). | Methods: Synthesis of Results and analysis |
|  | 13f | Describe any sensitivity analyses conducted to assess robustness of the synthesized results. | Methods: Synthesis of Results and analysis |
| Reporting bias assessment | 14 | Describe any methods used to assess risk of bias due to missing results in a synthesis (arising from reporting biases). | Methods: Certainty of Evidence assessment |
| Certainty assessment | 15 | Describe any methods used to assess certainty (or confidence) in the body of evidence for an outcome. | Methods: Certainty of Evidence assessment |
| **RESULTS** | | |  |
| Study selection | 16a | Describe the results of the search and selection process, from the number of records identified in the search to the number of studies included in the review, ideally using a flow diagram. | Results: Study selection; Figure 1 |
|  | 16b | Cite studies that might appear to meet the inclusion criteria, but which were excluded, and explain why they were excluded. | Results: Study selection; Figure 1 |
| Study characteristics | 17 | Cite each included study and present its characteristics. | Results: Table 1: Study Characteristics |
| Risk of bias in studies | 18 | Present assessments of risk of bias for each included study. | Results: Table 2: Summary of risk of bias assessment of included studies |
| Results of individual studies | 19 | For all outcomes, present, for each study: (a) summary statistics for each group (where appropriate) and (b) an effect estimate and its precision (e.g. confidence/credible interval), ideally using structured tables or plots. | Results: Table 1: Study Characteristics; Meta analysis: Figure 3, Figure 4, Figure 5 |
| Results of syntheses | 20a | For each synthesis, briefly summarise the characteristics and risk of bias among contributing studies. | Results: Table 1: Study Characteristics; Meta analysis: Figure 3, Figure 4, Figure 5.  Narrative synthesis, best evidence synthesis |
|  | 20b | Present results of all statistical syntheses conducted. If meta-analysis was done, present for each the summary estimate and its precision (e.g. confidence/credible interval) and measures of statistical heterogeneity. If comparing groups, describe the direction of the effect. | Meta-analysis: Figure 3, Figure 4, Figure 5 |
|  | 20c | Present results of all investigations of possible causes of heterogeneity among study results. | Meta-analysis: Figure 3, Figure 4, Figure 5 |
|  | 20d | Present results of all sensitivity analyses conducted to assess the robustness of the synthesized results. | N/A |
| Reporting biases | 21 | Present assessments of risk of bias due to missing results (arising from reporting biases) for each synthesis assessed. | Results: Certainty of evidence; Table 3 |
| Certainty of evidence | 22 | Present assessments of certainty (or confidence) in the body of evidence for each outcome assessed. | Results: Certainty of evidence; Table 3 |
| **DISCUSSION** | | |  |
| Discussion | 23a | Provide a general interpretation of the results in the context of other evidence. | Discussion |
|  | 23b | Discuss any limitations of the evidence included in the review. | Limitations |
|  | 23c | Discuss any limitations of the review processes used. | Limitations |
|  | 23d | Discuss implications of the results for practice, policy, and future research. | Recommendations |
| **OTHER INFORMATION** | | |  |
| Registration and protocol | 24a | Provide registration information for the review, including register name and registration number, or state that the review was not registered. | Registration |
|  | 24b | Indicate where the review protocol can be accessed, or state that a protocol was not prepared. | Registration |
|  | 24c | Describe and explain any amendments to information provided at registration or in the protocol. | N/A |
| Support | 25 | Describe sources of financial or non-financial support for the review, and the role of the funders or sponsors in the review. | Acknowledgments |
| Competing interests | 26 | Declare any competing interests of review authors. | Competing Interests |
| Availability of data, code and other materials | 27 | Report which of the following are publicly available and where they can be found: template data collection forms; data extracted from included studies; data used for all analyses; analytic code; any other materials used in the review. | N/A |

Table S9: PRISMA Abstract Checklist for Systematic Reviews
